# VIRMA mediates preferential m^6^A mRNA methylation in 3′UTR and near stop codon and associates with alternative polyadenylation

**DOI:** 10.1038/s41421-018-0019-0

**Published:** 2018-02-27

**Authors:** Yanan Yue, Jun Liu, Xiaolong Cui, Jie Cao, Guanzheng Luo, Zezhou Zhang, Tao Cheng, Minsong Gao, Xiao Shu, Honghui Ma, Fengqin Wang, Xinxia Wang, Bin Shen, Yizhen Wang, Xinhua Feng, Chuan He, Jianzhao Liu

**Affiliations:** 1 MOE Key Laboratory of Macromolecular Synthesis and Functionalization, Department of Polymer Science and Engineering, Zhejiang University, Hangzhou, Zhejiang 310027 China; 2 Department of Chemistry, Department of Biochemistry and Molecular Biology, Institute for Biophysical Dynamics, Howard Hughes Medical Institute, The University of Chicago, Chicago, IL 60637 USA; 3 College of Animal Sciences, Key Laboratory of Molecular Nutrition, Ministry of Education, Zhejiang University, Hangzhou, Zhejiang 310058 China; 4 State Key Laboratory of Reproductive Medicine, Department of Histology and Embryology, Nanjing Medical University, Nanjing, Jiangsu 210029 China; 5 Life Sciences Institute, Zhejiang University, Hangzhou, Zhejiang 310058 China

## Abstract

*N*^6^-methyladenosine (m^6^A) is enriched in 3′untranslated region (3′UTR) and near stop codon of mature polyadenylated mRNAs in mammalian systems and has regulatory roles in eukaryotic mRNA transcriptome switch. Significantly, the mechanism for this modification preference remains unknown, however. Herein we report a characterization of the full m^6^A methyltransferase complex in HeLa cells identifying METTL3/METTL14/WTAP/VIRMA/HAKAI/ZC3H13 as the key components, and we show that VIRMA mediates preferential mRNA methylation in 3′UTR and near stop codon. Biochemical studies reveal that VIRMA recruits the catalytic core components METTL3/METTL14/WTAP to guide region-selective methylations. Around 60% of VIRMA mRNA immunoprecipitation targets manifest strong m^6^A enrichment in 3′UTR. Depletions of *VIRMA* and *METTL3* induce 3′UTR lengthening of several hundred mRNAs with over 50% targets in common. VIRMA associates with polyadenylation cleavage factors CPSF5 and CPSF6 in an RNA-dependent manner. Depletion of CPSF5 leads to significant shortening of 3′UTR of over 2800 mRNAs, 84% of which are modified with m^6^A and have increased m^6^A peak density in 3′UTR and near stop codon after CPSF5 knockdown. Together, our studies provide insights into m^6^A deposition specificity in 3′UTR and its correlation with alternative polyadenylation.

## Introduction

Mammalian mRNA methylation *N*^6^-methyladenosine (m^6^A) sculpts the transcriptome in order to affect mRNA processing and metabolism, and represents a new mechanism to regulate gene expression analogous to epigenetic DNA and histone methylations^1–7^. The past 6 years have witnessed marked progress in m^6^A research, including the identification of m^6^A erasers^8, 9^, writers^10–13^, and readers^14–18^. The increase in the number of studies that explore the relationship between m^6^A effector proteins and their m^6^A transcripts have led to discoveries of m^6^A functions such as mRNA splicing^16^, export^19^, stability^14^, and translation^15, 17, 18, 20^.

The location of m^6^A sites on transcripts dictates m^6^A functions to a great extent. Transcriptome-wide m^6^A mapping of polyadenylated RNAs has revealed that the modification is highly enriched in 3′untranslated region (3′UTR) and near stop codon^21, 22^. The m^6^A modification is co-transcriptionally installed, and recent results indicated that m^6^A is preferentially added to the start of last exon of mRNAs^23^. The fundamental question with regard to why m^6^A is preferentially accumulated in 3′UTR and near the stop codon is still obscure. In addition, the 3′UTR m^6^A modification correlates with alternative polyadenylation (APA), a process that affects the inclusion of regulatory elements such as AU-rich elements, microRNA-targeting sites, and long non-coding RNA-binding sites, at 3′UTR to affect mRNA stability, translation, nuclear export, and cellular localization^24, 25^. We envision that the methyltransferase complex plays a decisive role to orchestrate the m^6^A modification patterns. The full methyltransferase complex itself has been predicted to have a molecular weight of more than 1000 kDa^26^ with factors that remain to be fully identified and characterized. Previous biochemical studies have identified METTL3/METTL14/WTAP as the catalytic core components of the human m^6^A methyltransferase complex^10, 11, 27^, among which METTL3 and METTL14 form a tight heterodimer in order to perform catalysis on a preferred motif sequence of GGACU^10^. No evidence suggests however that any of the three proteins control modification site specificity within cells.

Herein we report characterization of the full m^6^A methyltransferase complex in human HeLa cells and found that its component VIRMA mediates preferential mRNA methylation in 3′UTR and near the stop codon. VIRMA recruits the catalytic core components METTL3/METTL14/WTAP in order to guide region-selective methylations. Knockdown of VIRMA leads to loss of enrichment in 3′UTR and near the stop codon, and 3′UTR lengthening of certain group of mRNAs. VIRMA associates with polyadenylation cleavage factors in an RNA-dependent manner. Knockdown of the polyadenylation cleavage factor CPSF5 (CFIm25) results in significant shortening of 3′UTR of over 2800 mRNAs, 84% of which are modified with m^6^A and have increased m^6^A peak density in 3′UTR and near stop codon after CPSF5 knockdown. Together, our study provides insights into m^6^A deposition specificity in 3′UTR and its correlation with APA.

## Results

### Characterization of full m^6^A methyltransferase complex

**Fig. 1:**
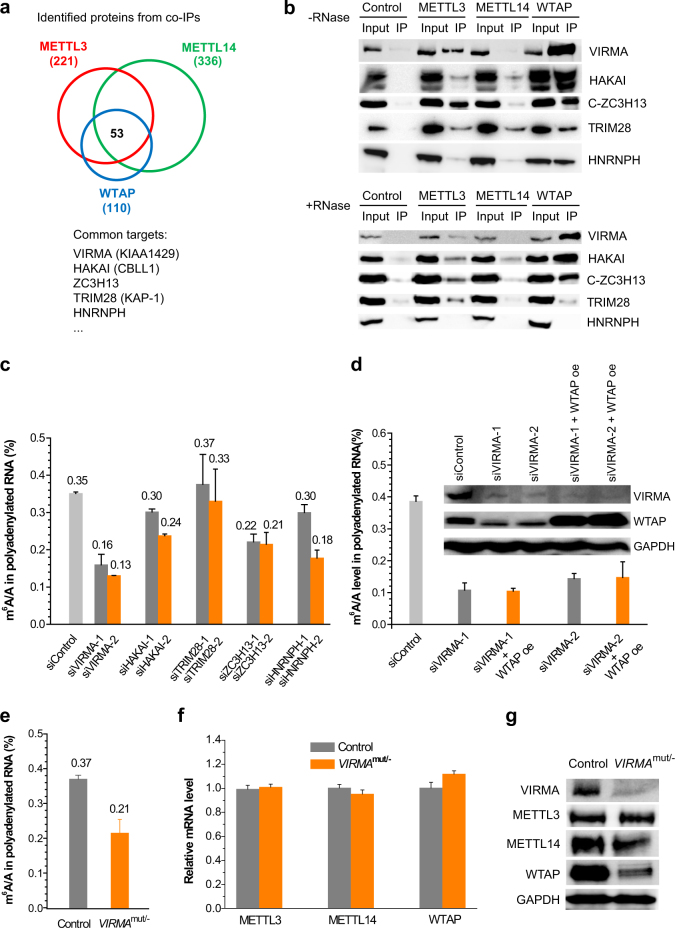
Proteomic identification of new m^6^A methyltransferase complex components and evaluation of their effects on mRNA m^6^A modification distribution. **a** Overlap of protein interactomes of METTL3, METTL14, and WTAP. Stable expression HeLa cells with dual-tagged (N-term tandem Flag and HA) METTL3, METTL14, and WTAP were subjected to tandem affinity purification with Flag and HA antibodies and further mass spectrometry identification. **b** Validation of selected common targets of METTL3, METTL14, and WTAP by western blotting. After Flag IP in the dual Flag-HA-tagged METTL3, METTL14, and WTAP stable cell lines, western blotting was performed to validate the interactions using endogenous antibodies. Because endogenous ZC3H13 antibody is not good for western blotting, Flag-tagged full-length ZC3H13 and its truncated forms N-terminal (N-ZC3H13) and C-terminal (C-ZC3H13) were overexpressed in the METTL3, METTL14, and WTAP stable cell lines for validation using Flag antibody after HA IP. **c** Effects of siRNA knockdown of VIRMA, HAKAI, ZC3H13, TRIM28, and HNRNPH on m^6^A level in HeLa cell line. Two different siRNA constructs were tested for each gene. Data are represented as means ± SEM of *n* = 3. *P* values calculated using one-tailed Student’s *t*-test between control and siRNA knockdown samples are <0.05, except for TRIM28 knockdown samples (*P* > 0.05). **d** Effects of VIRMA siRNA knockdowns on WTAP expression and polyadenylated RNA m^6^A levels. Overexpression (oe) of WTAP under VIRMA siRNA knockdowns cannot recover m^6^A level. Data are represented as means ± SEM of *n* = 3. *P* values calculated using one-tailed Student’s *t*-test between control and siRNA knockdown samples are <0.001, while *P* values between siVIRMA and siVIRMA/WTAP oe are larger than 0.05. **e** Comparison of m^6^A levels in polyadenylated RNAs in between control and *VIRMA*^mut/−^ HeLa cell lines. The *VIRMA*^mut/−^ HeLa cell line was generated by cas9 gene-editing system. Data are represented as means ± SEM of *n* = 3. *P* values calculated using one-tailed Student’s *t*-test between control and *VIRMA*^mut/−^ samples are <0.001. **f**, **g** Comparison of mRNA and protein expression levels of METTL3, METTL14, and WTAP between control and *VIRMA*^mut/−^ HeLa cell lines using RT-qPCR (**f**) and western blotting (**g**), respectively. GAPDH serves as control. *P* values calculated using one-tailed Student’s *t*-test between control and *VIRMA*^mut/−^ RT-qPCR samples are >0.05

To identify key components that can regulate m^6^A modification specificity, we began with a characterization of the full m^6^A methyltransferase complex. The co-immunoprecipitation (co-IP) assay was performed using the known core components METTL3/METTL14/WTAP as bait. HeLa cell lines stably expressing Flag and HA dual-tagged METTL3, METTL14, and WTAP were constructed, respectively^28^. Using a standard tandem affinity-based co-IP protocol, we purified and identified 221, 336, and 110 proteins by mass spectrometry from METTL3, METTL14, and WTAP co-IP products, respectively, revealing an overlap of 53 proteins (Fig. 1a and Table S1). By methodical study of these potential targets, we narrowed down and picked up several potential candidates from the nucleus in order to validate proteomic interactions using western blotting, including VIRMA (KIAA1429, ~202 kDa), HAKAI (Cbl photo oncogene like 1, also termed CBLL-1, ~55 kDa), ZC3H13 (zinc finger CCCH-type containing 13, ~197 kDa), TRIM28 (tripartite motif containing 28, also termed KAP-1, ~89 kDa), HNRNPH (heterogeneous nuclear ribonucleoprotein H, ~49 kDa), HNRNPK (heterogeneous nuclear ribonucleoprotein K, ~51 kDa), and HNRNPU (heterogeneous nuclear ribonucleoprotein U, ~91 kDa; Fig. 1b and Fig. S1a). Among these targets, VIRMA, HAKAI, and ZC3H13 were reported to closely interact with WTAP^29, 30^^.^ The results suggested interactions between VIRMA/HAKAI/ZC3H13/TRIM28/HNRNPH and core catalytic components of METTL3/METTL14/WTAP, while HNRNPK and HNRNPU were not seen in the IP products (Fig. 1b and Fig. S1a). HNRNPH reveals RNA-dependent interactions with the core factors (Fig. 1b). Because the current commercial endogenous ZC3H13 antibody was not good enough for western blotting, we cloned tagged ZC3H13 and its truncated forms. The result showed that C-terminal ZC3H13 (C-ZC3H13) interacted with METTL3 and WTAP. In addition, these validated targets were more enriched in WTAP IP.

### Screening and identification of key methyltransferase components that affect mRNA m^6^A modification

We next utilized siRNA screening in order to check whether these potential methyltransferase components are able to affect the levels of mRNA m^6^A. Using two distinct siRNAs for each gene (Fig. 1c and Fig. S1b-f), we found that knockdown of VIRMA, HAKAI, and ZC3H13 led to significant and consistent decreases of the total m^6^A levels in polyadenylated RNAs by approximately 59%, 23%, and 39%, respectively, while TRIM28 knockdown did not result in noticeable change of m^6^A (Fig. 1c). For HNRNPH, two different siRNAs induced m^6^A decreases by 14% and 49%, respectively. The depletion of VIRMA led to the biggest reduction of the total m^6^A level among all the tested components, including METTL3, METTL14, and WTAP, which exhibited about 30%, 40%, and 50% decreases in m^6^A levels, respectively^10^. These observations are consistent with previous reports that VIRMA is a key member of the m^6^A methyltransferase complex and is required for mRNA methylation^11, 13^. In *Drosophila*, VIRMA is required for viability and is involved in the female-specific splicing of Sxl transcripts^31^, whereas the function of its human VIRMA homolog remains unknown. Mammalian VIRMA is a large protein (202 kDa) and the largest known component within the methyltransferase complex. VIRMA siRNA knockdown led to significant reduction of WTAP, however, overexpression of WTAP cannot rescue the m^6^A level (Fig. 1d). We suspect that VIRMA might serve as a scaffold of the methyltransferase complex and play roles in bridging the catalytic core components and RNA substrates in order to affect the installation of m^6^A at specific locations.

Next, we employed CRISPR-cas9 genome editing in order to generate *VIRMA*-depleted HeLa cell line following the published protocol^32^. It turned out that we could only obtain the partial knockout *VIRMA*^mut/−^ cells in multiple trials, which indicates that VIRMA is essential for cell viability. With the *VIRMA*^mut/−^ mutant cell line created (Fig. S1g), we isolated poly(A)-tailed mRNA, measured the m^6^A level, and found a ~43% reduction of the m^6^A level compared to the control cell line (Fig. 1e and Fig. S1h), consistent with the siRNA knockdown result. We further checked whether VIRMA depletion would disturb expressions of METTL3, METTL14, and WTAP. The quantitative PCR (qPCR) experiment displayed no noticeable changes of their mRNA expressions (Fig. 1f), while the protein level of WTAP was dramatically reduced (Fig. 1g), corroborating the siRNA knockdown result (Fig. 1d). Consistent with the western blotting data, the immunofluorescence staining experiment showed a much weaker WTAP signal in *VIRMA*^mut/−^ than the control cell line (Fig. S1i), thus suggesting that VIRMA stabilizes WTAP inside the nucleus.

### VIRMA mediates mRNA m^6^A methylation in 3′UTR and near stop codon

The major impact of VIRMA on the total m^6^A level on mRNA prompted us to investigate the positions of m^6^A loss along mRNA transcripts. We applied methylated RNA immunoprecipitation followed by next-generation high-throughput sequencing (m^6^A-seq or MeRIP-seq)^3, 21^ in order to characterize m^6^A site distribution in siVIRMA, *VIRMA*^mut/−^, and their corresponding control HeLa cell lines. Following the published protocol^33^, poly(A)-selected RNA was fragmented and immunoprecipitated using anti-m^6^A antibody. Libraries from both input and IP RNA fragments in two replicates were prepared and subjected to massive parallel sequencing with different sequencing depth (Table S2 and Fig. S2a-d). Reads were uniquely aligned to a reference transcriptome and a stringent cutoff threshold for false discovery rate (FDR) of <0.05 was used in order to call high-confidence m^6^A peaks. For instance, the bioinformatics analysis yielded 11 086 m^6^A peaks in coding gene transcripts and long non-coding transcripts for control cells, and 13 125 m^6^A peaks for *VIRMA*^mut/−^ cells (Table S3). In between the two replicates, the control cell line has around 60–71% of m^6^A peaks in common with the *VIRMA*^mut/−^ cell line, corresponding to 65–78% overlap in gene transcripts.

**Fig. 2:**
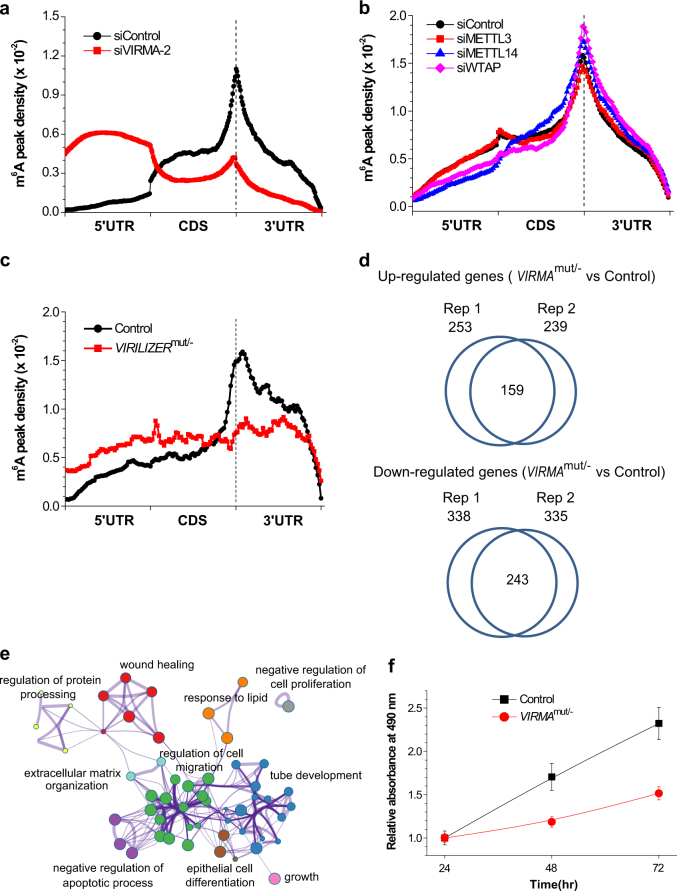
VIRMA affects cellular functions. **a**, **b** Effects of depletion of VIRMA (**a**) and METTL3, METTL14, and WTAP (**b**) by siRNA knockdowns on the profiles of m^6^A peak density along mRNA transcript. **c** Effect of partial *VIRMA* knockout (*VIRMA*^mut/−^) on the profiles of m^6^A peak density along mRNA transcript. **d** Differentially expressed genes (fold change > 2) were identified by the RNA-seq in between *VIRMA*^mut/−^ and control cell lines. The number of overlapped upregulated and downregulated genes are shown. **e** Gene ontology (GO) and enrichment analysis of the differentially expressed and m^6^A-containing genes. **f** Estimate of *VIRMA* depletion on cell proliferation by MTS assay. Data are represented as means ± SEM of *n* = 3

We then examined the m^6^A peak distribution along the transcripts. Each m^6^A peak was assigned to one of three non-overlapping transcript segments, including 5′UTR, coding sequence (CDS), and 3′UTR, and then normalized by the length of its located segment. As typical, control cells display an obvious enrichment of m^6^A in 3′UTR and near stop codon, whereas this signature enrichment disappears in the siVIRMA and *VIRMA*^mut/−^ cells with the whole profile becoming relatively flat (Fig. 2a, c), suggesting a significant reduction of m^6^A modification in 3′UTR and near stop codon regions. We checked our previously published m^6^A-seq data under siRNA knockdowns of METTL3, METTL14, and WTAP in order to examine how these affect the distribution of the m^6^A peak along the transcript^10^. We did not observe noticeable distribution pattern changes (Fig. 2b), indicating that the depletion of catalytic units of the methyltransferase complex reduces m^6^A installation evenly at most regions of mRNAs. We also depleted HAKAI and ZC3H13 to see their influences on m^6^A distribution along mRNA transcripts and found a slight decrease of m^6^A peak density in 3′UTR and near stop codon (Fig. S2e and f), which is not as obvious as that of VIRMA depletion. Meanwhile, the m^6^A enrichment in long exons exhibited no change, showing 82% versus 84% of exon m^6^A peaks in exons longer than 400 nucleotides for control and *VIRMA*^mut/−^ cells, respectively. Together, VIRMA plays a role in mediating selective m^6^A deposition 3′UTR and near stop codon of mRNAs.

### VIRMA affects cellular functions

Differentially expressed (DE) genes identified from RNA-seq between control and *VIRMA*^mut/−^ cell lines were analyzed. Based on two replicate samples, there are 159 upregulated and 243 downregulated genes with an expression change of more than twofolds, up to 66% of which are m^6^A-modified (Fig. 2d and Table S4). Close inspection of m^6^A-containing DE genes revealed that the m^6^A abundance inversely correlates with the expression level. The genes having decreased m^6^A level show increased expression and vice versa, which could be explained by the decay role of m^6^A^14^. Obviously, VIRMA influences a substantial set of mRNA transcripts. Gene ontology analysis of the m^6^A-containing DE genes yielded the enrichment network of cellular functions (Fig. 2e and Table S5). Notable gene clusters include negative regulation of cell proliferation, cell growth, negative regulation of the apoptotic process, regulation of cell migration, and tube development, indicating that VIRMA regulates basic cellular processes. When we generated the *VIRMA*^mut/−^ cell line, we found that the cell proliferation is notably reduced compared to the control cell line (Fig. 2f). This result is in agreement with the fact that some of the genes, *IGFBP5* and *NTOCH1* for instance, which are involved in the cluster of negative regulation of cell proliferation, are markedly upregulated after *VIRMA* depletion.

### Validation of VIRMA-regulated m^6^A genes identified by high-throughput sequencing

**Fig. 3:**
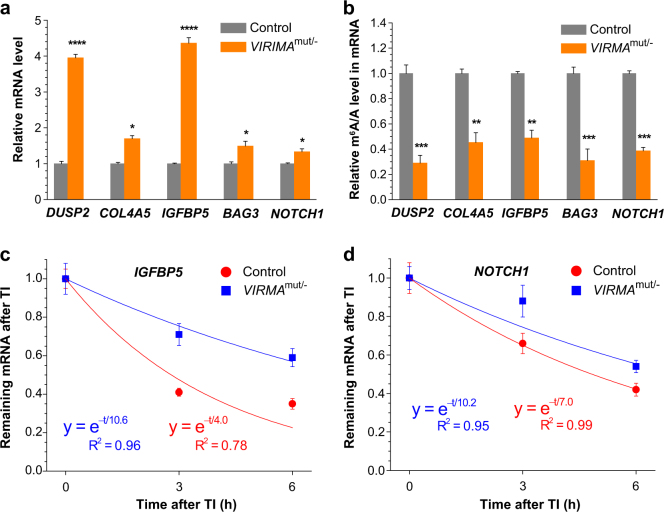
VIRMA modulates abundance and lifetime of mRNAs through mediating m^6^A installation. **a**,**b** Validation of selected mRNA targets with upregulated expression (**a**) and decreased m^6^A level (**b**) upon *VIRMA* depletion in HeLa cell line. *P* values calculated using one-tailed Student’s *t*-test between control and *VIRMA* depletion samples; *P < 0.05, **P < 0.01, ***P < 0.001, ****P < 0.0001. Data are represented as means ± SEM of *n* = 3. **c**, **d** Measurement of lifetimes of selected mRNAs *IGFBP5* (**c**) and *NOTCH1* (**d**). Data are represented as means ± SEM of *n* = 3

We next used experimental examples in order to validate the high-throughput RNA-seq and m^6^A-seq results and understand how VIRMA affects gene expressions by modulating m^6^A modification. As revealed by RNA-seq and m^6^A-seq, we selected several m^6^A-containing genes, including *DUSP2*, *COL4A5*, *IGFBP5*, *BAG3*, and *NOTCH1* (Table S6), which showed increased expression by 1.5- to 4.5-folds (Fig. 3a). We isolated mRNAs (input), subjected them to anti-m^6^A antibody IP, saved the flow through (FT), and quantified each gene in input, FT, and IP samples by RT-qPCR. The m^6^A levels of these genes dropped by 50–70%, respectively (Fig. 3b), which is in agreement with decreased m^6^A peak density in the m^6^A-seq result. We further utilized the RNA decay assay in order to substantiate the inverse correlation of mRNA m^6^A abundance with expression level. We measured mRNA levels of *IGFBP5* and *NOTCH1* at 3 and 6 h post transcription inhibition (TI) and calculated their lifetimes. As expected, these mRNAs in *VIRMA*^mut/−^ cells showed longer lifetimes by 3.2–6.6 h than those in control cells (Fig. 3c,d). These examples are in agreement with the m^6^A-mediated mRNA decay theory.

### VIRMA guides m^6^A modification at specific site

**Fig. 4:**
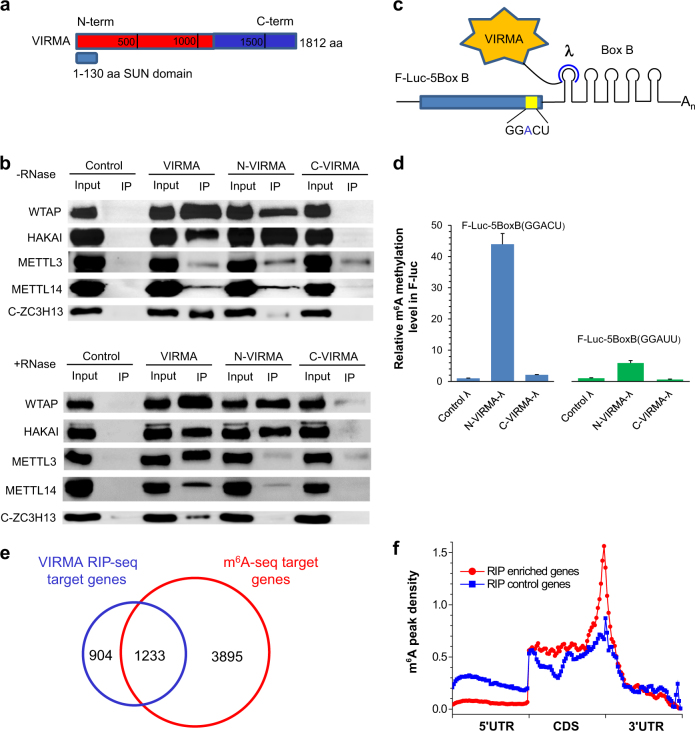
VIRMA recruits catalytic components METTL3/METTL14/WTAP in order to guide mRNA m^6^A methylation at specific site. **a** Schematic of domain architecture (aa, amino acids) of VIRMA, N terminus of VIRMA (N-VIRMA, aa 1–1130), and C terminus of VIRMA (C-VIRMA, aa 1131–1812). **b** Co-immunoprecipitation experiments in HeLa cells in order to dissect interactions of different domains of VIRMA with other methyltransferase components. RNase was added in order to determine if the interaction was RNA-dependent. HSV-tagged C-ZC3H13 was co-expressed for immunoprecipitation experiment and corresponding HSV-tag antibody was used in the western blotting. **c** Construct of the tethering reporter assay. The mRNA reporter consists of a firefly luciferase sequence as the coding region and five Box B sequence at 3′UTR (F-Luc-5BoxB). There exists a GGACU motif near the stop codon. Both N-VIRMA and C-VIRMA were fused with λ peptide (N-VIRMA-λ and C-VIRMA-λ), which recognizes Box B RNA with a high affinity. **d** Measurement of m^6^A level of F-Luc-5BoxB mRNAs under co-expression with different truncated forms of VIRMA. The synonymously mutated construct with GGAUU was tested for comparison. *Renilla* luciferase was used as an internal control to normalize the F-Luc signal. **e** Overlap of VIRMA RIP-seq mRNA targets with m^6^A-seq targets in HeLa cells. Endogenous VIRMA antibody was used for direct RNA immunoprecipitation. RIP-seq-enriched genes were defined as FDR ≤ 0.05 and log_2_(IP/Input) ≥ 1. **f** Comparison of m^6^A peak density profiles between RIP-seq-enriched and control genes. RIP-seq control genes (1909) were defined as FDR ≤ 0.05 and log_2_(IP/Input) < 1

After the in vivo experimental to examine VIRMA-regulated m^6^A modification, we proceeded to study the VIRMA protein. Human VIRMA has a full length of 1812 amino acids (aa) with two isoforms. The isoform 2 has a length of 1147 aa and possesses the same N-terminal 1130 aa as the full-length VIRMA. We defined isoform 2 as N-VIRMA and 1131–1812 aa region as C-VIRMA (Fig. 4a). N-VIRMA has a 130 aa SUN domain at the beginning. We subcloned Flag-tagged full-length *VIRMA* and its truncated forms into modified pTriEx 1.1-Neo vector and successfully expressed them in mammalian cells (Fig. S3). After overexpression and co-IP of VIRMA, N-VIRMA, and C-VIRMA in HeLa cells, western blotting was performed in order to understand which part of VIRMA interacts with core methyltransferase components. The result reveals that the VIRMA is engaged in binding WTAP/HAKAI/ZC3H13/METTL3/METTL14 in an RNA-independent manner, with its N-terminal showing a major role to recruit catalytic core members METTL3/METTL14/WTAP (Fig. 4b).

We further performed a tethering assay from HeLa cells in order to support that N-VIRMA could recruit core methyltransferase components. N-VIRMA or C-VIRMA was fused with a λ peptide, which specifically recognizes Box B RNA with a high affinity^34^ (Fig. 4c). A Five Box B sequence was inserted into the 3′UTR of firefly luciferase to form an F-Luc-5BoxB construct, which features an upstream GGACU motif 73 bp away from the stop codon. We found that tethering N-VIRMA-λ to F-Luc-5BoxB led to around 22- to 44-fold higher m^6^A modification levels than C-VIRMA-λ and λ, respectively (Fig. 4d). When the motif GGACU was synonymously mutated into GGAUU, the m^6^A modification level of F-Luc-5BoxB was dramatically decreased and only a small difference was observed between N-VIRMA-λ, C-VIRMA-λ, and λ constructs. Together, these results confirm that VIRMA is able to guide specific m^6^A modification inside cells.

### VIRMA-associated mRNAs have a sharp m^6^A density peak in 3′UTR and near stop codon

Next, we asked whether VIRMA-associated mRNA transcripts are enriched with m^6^A in 3′UTR and near stop codon. The RNA immunoprecipitation sequencing (RIP-seq) experiment using endogenous anti-VIRMA antibody identified 2137 transcripts with enrichment greater than twofolds, 1233 of which overlap with m^6^A-seq targets (Fig. 4e and Table S7). We then calculated m^6^A peak density profiles of VIRMA-associated mRNA transcripts versus RIP control targets with enrichment less than twofolds, and found that the former group presented a sharp m^6^A density peak in 3′UTR around the stop codon, indicating a higher m^6^A enrichment than the control within this region (Fig. 4f). The RIP-seq result supports VIRMA as a key methyltransferase component to bind RNA substrates and mediate m^6^A modification in 3′UTR and near stop codon.

### VIRMA associates with polyadenylation cleavage factors and methyltransferase components affect APA

**Fig. 5:**
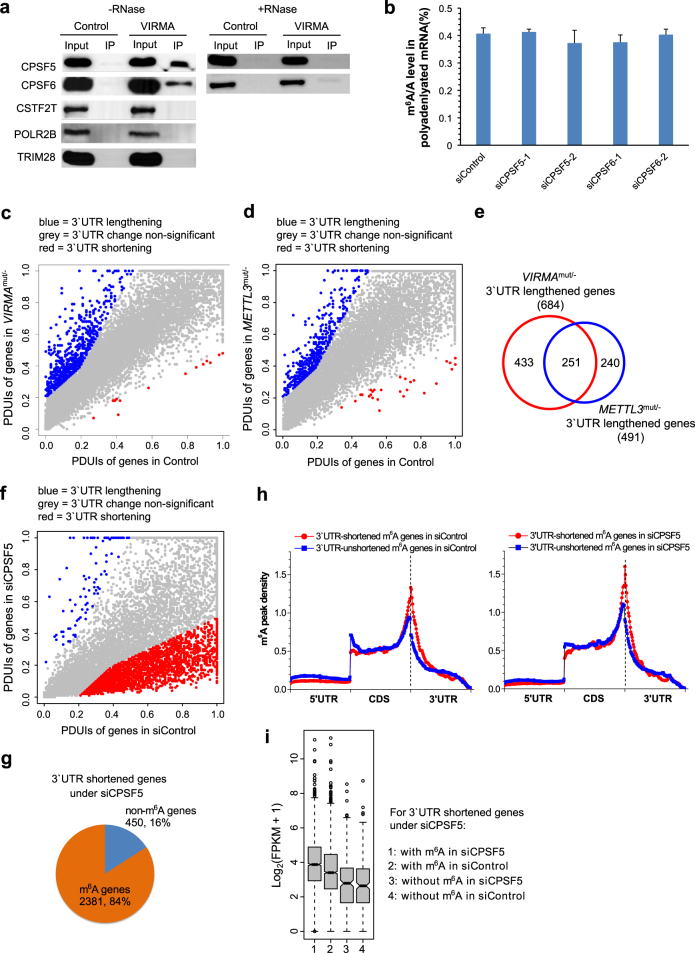
VIRMA associates with polyadenylation cleavage factors. **a** Western blots to validate potential proteomic targets of VIRMA, including polyadenylation cleavage factors CPSF5 and CPSF6, cleavage stimulation factor CSTF2T, poly(A)-binding protein PABP1, RNA polymerase II subunit POLR2B, and transcriptional intermediary factor TRIM28. **b** Effect of siRNA knockdown of CPSF5 and CPSF6 on m^6^A level of polyadenylated RNAs. *P* values calculated using one-tailed Student’s *t*-test between control and knockdown samples are >0.05. Data are represented as means ± SEM of *n* = 3. **c**, **d** Scattering plots of PDUIs in between control and *VIRMA* (**c**) and *METTL3* (**d**) mutant cells, where 3′UTRs of mRNAs are significantly shortened or lengthened with FDR ≤ 0.05, absolute ΔPDUI ≥ 0.2, and at least twofold change of PUDIs. **e** Overlap of 3′UTR-lengthened genes regulated by *VIRMA* and *METTL3* depletion effects. **f** Scattering plots of PDUIs in between siControl and siCPSF5 cells, where 3′UTRs of mRNAs are significantly shortened or lengthened with FDR ≤ 0.05, absolute ΔPDUI ≥ 0.2, and at least twofold change of PUDIs. **g** Pie chart shows percentages of m^6^A and non-m^6^A genes with 3′UTR shortening under the CPSF5 knockdown. Eighty-four percent of shortened genes are enriched with m^6^A modification. Chi-square test gives a *P* value < 1e-16. **h** The m^6^A peak density profiles of m^6^A genes in HeLa siControl and siCPSF5 cells grouped by their 3′UTR shortening induced by CPSF5 knockdown. **i** Differential gene expressions for 3′UTR-shortened m^6^A and non-m^6^A genes, respectively, after CPSF5 knockdown. RPKM stands for reads per kilobase per million mapped reads

Nuclear mRNA m^6^A installation is a co-transcriptional event. We performed a proteomic study of VIRMA-bound proteins. We identified potential partners including polyadenylation cleavage factors CPSF5 and CPSF6 (CFIm68), cleavage stimulation factor CSTF2T, RNA polymerase II subunit POLR2B, and transcriptional intermediary factor TRIM28 (Table S1). We noted that VIRMA associated with CPSF5 and CPSF6 in an RNA-dependent manner (Fig. 5a). It is known that CPSF5 and CPSF6 form a tight tetramer complex named CFIm and preferentially binds a UGUA motif^35–37^, which is typically located 40–100 nucleotides upstream of the poly(A) site (PAS). This sequence motif and its binding by CPSF5 and CPSF6 affect APA of mRNAs in order to alter their 3′UTR lengths. According to the model of mRNA cleavage and polyadenylation^24, 25^, CFIm complex (CPSF5 and CPSF6) and cleavage stimulation factors (e.g., CSTF2T) bind upstream and downstream of PAS, respectively. In principle, the enriched m^6^A modification sites in 3′UTR and near stop codon are close to the upstream CFIm-binding sites, supporting the observed association of methyltransferase complex with polyadenylation cleavage factors.

We proceeded to study the relationship between the m^6^A methylation and CPSF cleavage factors. First, we investigated effects of CPSF5 and CPSF6 depletion on the total mRNA m^6^A level. When each gene was knocked down using two distinct siRNAs, m^6^A levels were almost unchanged (Fig. 5b and Fig. S4a), respectively, indicating that 3′UTR polyadenylation may occur after m^6^A methylation and thus does not affect m^6^A deposition.

Next, we analyzed mRNA APA between *VIRMA*^mut/−^ and control cells using a bioinformatics algorithm DaPars^38^. The alteration of the 3′UTR length was quantified as a change in percentage of distal PAS usage index (ΔPDUI), which was used to identify lengthening (positive index versus control) or shortening (negative index versus control) within 3′UTR. The result revealed 684 genes possessing 3′UTR lengthening versus 13 genes with 3′UTR shortening after the depletion of *VIRMA* (Fig. 5c and Table S8), indicative of a switch to increased distal PAS usage. We also generated a *METTL3*^mut/−^ HeLa cell line by using CRISPR-cas9 (Fig. S4b and c) in order to examine differences of the 3′UTR length upon depletion of METTL3. Similarly, 491 genes were shifted to distal PAS usage and possessed longer 3′UTR, while only 25 genes showed 3′UTR shortening (Fig. 5d). There was an overlap of 251 targets having 3′UTR lengthening between *VIRMA*- and *METTL3*-affected genes (Fig. 5e). We selected targets to validate the mRNA 3′UTR lengthening effect in each cell line, including *E2F5*, *SMNDC1*, *UBE2V2*, *NBN*, and *TBPL1* for *VIRMA*^mut/−^, and *VPK1*, *SMNDC1*, *UBE2V2*, and *ZRANB2* for *METTL3*^mut/−^, respectively (Fig. S4d and e). As expected, all these targets exhibited increased distal PAS usage. Taken together, these observations indicate that the role of m^6^A methyltransferase components is to facilitate the selection of proximal polyadenylation sites within 3′UTR of mRNAs.

### CPSF5 knockdown shortens 3′UTR of m^6^A-rich mRNAs

As previously reported, CPSF5 knockdown greatly influences APA and induces 3′UTR shortening of a significant number of mRNAs^38^. Among identified 2831 genes with 3′UTR shortening under siRNA knockdown of CPSF5, 84% targets are m^6^A-modified (Fig. 5f, g and Table S8). If the m^6^A methylome in normal HeLa cell is divided into two groups, namely CPSF5-regulated 3′UTR-shortened and -unshortened m^6^A genes, the former group displays a much higher m^6^A peak density in 3′UTR and near stop codon over the latter group (Fig. 5h). Another finding is that compared to CPSF5-regulated non-m^6^A genes, m^6^A genes show more obviously increased peak density in 3′UTR and near stop codon after CPSF5 knockdown (Fig. 5h).

The knockdown of CPSF5 mostly results in shortening of 3′UTR of m^6^A-containing mRNAs, which are expected to affect gene expression. We next analyzed mRNA expression of 3′UTR-shortened genes regulated by CPSF5. As previously reported, 3′UTR shortening caused by CPSF5 knockdown reduces microRNA- and AU-rich element-mediated gene repression and thus leads to elevated mRNA expressions^38^. We compared m^6^A and non-m^6^A gene expressions in both control and CPSF5 knockdown cells (Fig. 5i). We noticed two features: (i) m^6^A-containing genes showed up to twofold higher expression in general than non-m^6^A genes in both control and knockdown cells; and (ii) m^6^A-containing genes showed more dramatic upregulation after CPSF5 knockdown while non-m^6^A genes only exhibited slight increases in expression. We selected several target genes, including *TACC2*, *SPEN*, *NOTCH1*, and *PPPIR13B* in order to validate high-throughput sequencing data. Indeed, all the tested genes underwent 3′UTR shortening and increased expression with CPSF5 knockdown (Fig. S4f and g).

**Fig. 6:**
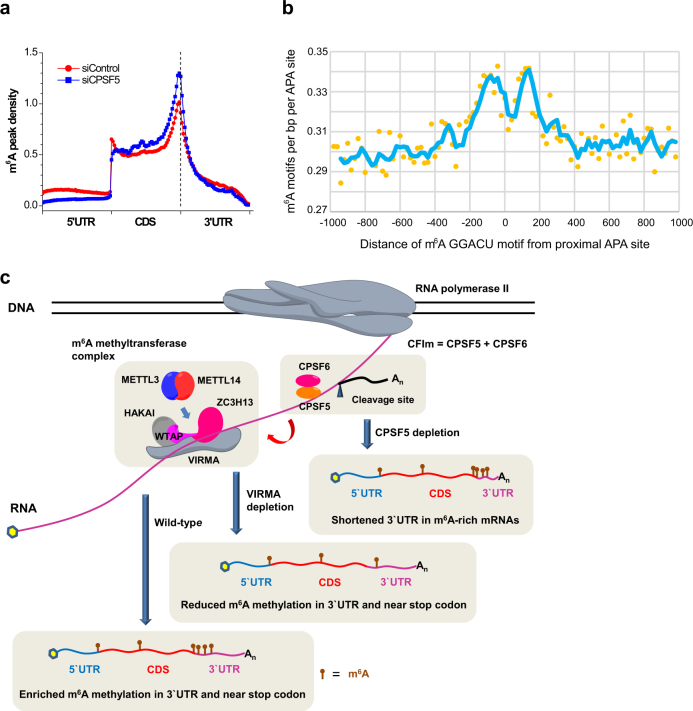
Correlation of 3′UTR m^6^A methylation with alternative polyadenylation. **a** The m^6^A peak density profiles of m^6^A genes in HeLa siControl and siCPSF5 cells. **b** m^6^A GGACU motif coverage versus motif distance from proximal APA site along mRNA transcript. **c** Proposed model for VIRMA-regulated m^6^A methylation specificity in 3′UTR and near stop codon and correlation of m^6^A methylation with APA

We also observed that the m^6^A methyltransferase complex and CFIm complex possess a substantial overlap of targets. VIRMA and CPSF5 are major components from both complexes, respectively, and 43% of VIRMA RIP-seq m^6^A genes overlap with CPSF5-regulated ones. Globally, CPSF5 knockdown leads to an increase of m^6^A peak density in 3′UTR and near stop codon (Fig. 6a), possibly due to unchanged m^6^A level and shortened 3′UTR length of partial m^6^A genes. We further checked the distance between proximal APA sites and m^6^A methylation sites. The plot of m^6^A motif GGACU coverage indicated that they are in close vicinity in 3′UTR of mRNAs (Fig. 6b), indicating potential cross talk between proximal APA and m^6^A methylation sites.

## Discussion

The specificity of the m^6^A modification in 3′UTR and near stop codon in mammalian mRNAs has been a long-standing puzzle in the field, despite increased efforts on the part of researchers and their discoveries about the cellular functions of m^6^A over the past few years. The location of m^6^A on mRNA or its modification pattern has been closely linked with downstream RNA metabolism. For example, YTHDF2, an m^6^A reader protein, binds around 1300 high-confident m^6^A-containing mRNAs and has a role to mediate their decay^14^. In total, around 56% of YTHDF2-binding sites are located in 3′UTR and near stop codon. A disruption of site-specific methylation could induce major effects for downstream mRNA metabolism.

It is known that catalytic core components METTL3/METTL14 have substrate sequence specificity to GGACU motif^10^, but only a small portion of consensus sites across mRNA transcriptome have been methylated with a particular enrichment in 3′UTR and near stop codon. It is thus reasonable to speculate that certain factors within the methyltransferase complex dictate METTL3/METTL14 in order to orchestrate the observed methylation pattern on mRNA. The methyltransferase complex has an estimated size larger than 1000 kDa^26^, in which METTL3/METTL14/WTAP occupy ~200 kDa. Based on proteomic results and biochemical validation, we listed additional components of the m^6^A methyltransferase complex that include VIRMA (~202 kDa), HAKAI (~55 kDa), ZC3H13 (~197 kDa), TRIM28 (~89 kDa), and HNRNPH (~49 kDa), the latter two of which need to be further validated.

We show that VIRMA plays a role in mediating mRNA m^6^A methylation in 3′UTR and near stop codon. In the VIRMA-depleted cell lines, the signature pattern of m^6^A enrichment in 3′UTR and near stop codon disappears, indicating a significant loss of m^6^A modification around this region. We propose a model that VIRMA may serve as a scaffold to hold WTAP/HAKAI/ZC3H13 together creating a suitable pocket to accommodate METTL3/METTL14 mainly through WTAP in order to guide m^6^A modification in 3′UTR around the stop codon (Fig. 6c). Once VIRMA is depleted, access of METTL3/METTL14 to specific mRNA substrates is significantly reduced (as indicated by the noticeable reduction of the global mRNA methylation with VIRMA knockdown), and m^6^A modification specificity is lost. Transcriptome-wide m^6^A mapping also showed m^6^A enrichment on the long internal exon of mRNAs^21^; based on our study VIRMA does not appear to contribute to this enrichment. One future interesting study could focus on the mechanism involved in controlling m^6^A specificity on long exons in the future.

We probed the correlation of m^6^A methylation with 3′UTR APA^23, 39^. The methyltransferase component VIRMA associates with the polyadenylation CFIm complex in an RNA-dependent manner and the m^6^A consensus motif is close to the proximal APA site within 3′UTR. Depletions of *VIRMA* and *METTL3* lead to 3′UTR lengthening of mRNA transcripts, which suggests that the methyltransferase complex facilitates proximal APA. This is consistent with an early work that methylated transcripts tend to be coupled with the proximal APA site and thus have shortened 3′UTRs^39^. Up to 84% of 3′UTR-shortened mRNA transcripts induced by CPSF5 knockdown are m^6^A-modified, indicating that CPSF5 and m^6^A methyltransferase complex work on a large number of targets in common. In particular for these common targets, knockdown of CPSF5 gives rise to a higher m^6^A density in 3′UTR and near stop codon region. It has been proposed that two UGUA elements located upstream and downstream of a proximal PAS are bound by CPSF5 in CFIm complex, which loops out the proximal PAS to skip cleavage. The methyltransferase complex may work on RNA sequences nearby in order to suppress the looping effect and promote the selection of proximal PAS by the cleavage and polyadenylation machinery. Future work could reveal causal relationship between specific m^6^A methylation and APA.

It has been reported that 3′UTR shortening of mRNAs induced by CPSF5 depletion leads to enhanced cellular proliferation and tumorigenicity through elevated expression of growth-promoting factors in glioblastoma^38^. Loss of intrinsic microRNA sites and AU-rich elements within 3′UTR was thought to result in the expression upregulation. When we separate contributions of m^6^A-containing genes and non-m^6^A genes to overall expression upregulation, however, we found the former group dominates over the latter one. It will be interesting to look into what interplay exists between m^6^A and other regulatory elements in 3′UTR in the regulation of mRNA expression.

To conclude, this work extends our understanding of mammalian mRNA m^6^A modification specificity in 3′UTR and near stop codon, and manifests the correlation of m^6^A methylation with APA. The work reveals the underlying mechanism through VIRMA that recruits the methyltransferase core components and interacts with polyadenylation cleavage factors CPSF5 and CPSF6. The discovery implies the cross talk between the machineries of m^6^A methylation and polyadenylation during mRNA processing, suggestive of its functional relevance in the regulation of mRNA metabolism.

## Materials and methods

### Cell culture, siRNA knockdown, and plasmid transfection

Human HeLa cell line was grown in Dulbecco’s modified Eagle’s medium (DMEM)/high-glucose media (HyCloneSH30243.01) supplemented with 10% fetal bovine serum (FBS; Gibco 10270) and 1% 100× Pen Strep (HyClone SV30010). HEK 293T was grown in DMEM/high-glucose media (HyCloneSH30027.01) and was only used in the protein expression test. All the siRNAs were ordered from Qiagen with sequences shown in Table S9. All the antibodies used in this work were listed in Table S10. Transfection was achieved by using Lipofectamine RNAiMAX (Invitrogen) for siRNA, or Lipofectamine 2000 (Invitrogen) for the plasmid following the manufacturer’s protocols.

### Cloning of VIRMA

The *VIRMA* N-terminal (*VIRMA* isoform 2) cDNA was purchased from GE Dharmacon (MHS6278-211688919, *KIAA1429* isoform 2); *VIRMA* C-terminal (1131–1812 aa) cDNA was synthesized by and purchased from GenScript (see Supplementary Information). The *VIRMA* N-terminal and C-terminal were first cloned into mammalian vector pcDNA3 (Invitrogen) with an N-terminal Flag tag, respectively, while the full length of *VIRMA* was constructed by Gibson assembly (New England Biolabs) of N-terminal and C-terminal cDNA sequences.

The plasmids containing *VIRMA*, T2A peptide (EGRGSLLTCGDVEENPGP), and enhanced green fluorescent protein (eGFP) in a sequential manner in pcDNA3 were constructed by Gibson assembly in order to monitor the VIRMA protein expression by fluorescence signal of eGFP. A widely used sequence containing T2A peptide and eGFP was first subcloned into pcDNA3 by Gibson assembly. Primers are listed in Table S9. The vector generated above contains a Kozak consensus (5′-ACCATG-3′), immediately followed by 3× Flag, T2A, and eGFP as an indicator for protein expression. The vector contains two unique restriction sites, which are *Nco*I at the Kozak consensus, and *Age*I immediately after the stop codon of eGFP. Three forms of *VIRMA* (full-length, N-terminal, and C-terminal) were cloned into the recombinant pcDNA3 vector above using Gibson assembly, with a 2× GGGGS spacer in between each form of *VIRMA* and 3× Flag tag. Primers are listed in Table S9.

Due to low expression level of *VIRMA* inside mammalian cells using pcDNA3 vector, all three forms of *VIRMA*, including full-length, N-terminal, and C-terminal followed by 3× Flag, T2A, and eGFP, were subcloned into pTriEx 1.1-Neo (Novagen) using restriction sites of *Nco*I and *Age*I. These plasmids were used in experiments related to VIRMA protein expression.

### Cloning of ZC3H13

The full-length, N-terminal (N-*ZC3H13*, 1–1106 aa), and C-terminal (C-*ZC3H13*, 1107–1669 aa) of *ZC3H13* (see Supplementary Information) were subcloned into a modified version of pTriEx 1.1-Neo (with a strong translational enhancer sequence SP163 added right before the start codon ATG, a 2× GGGGS spacer and a 3× Flag tag followed by a T2A sequence and eGFP as indicator at C-terminal near stop codon TAA) with restriction sites *Bam*HI and *Age*I. HA-tagged forms of *ZC3H13* (full-length, N-*ZC3H13* and C-*ZC3H13*) were constructed in the same way (3× Flag tag replaced by a HA tag). The used primers were listed in Table S9.

### Generation of *VIRMA*^mut/−^ cell line using CRISPR-cas9 editing system

We followed the published protocol^32^. The cas9-targeted sequence and editing result of *VIRMA* gene were shown in Fig. S1g.

### RNA isolation

Total RNA was isolated from cells with TRIZOL reagent (Invitrogen). Polyadenylated RNA was extracted using Genelute mRNA miniprep kit (Sigma), followed by removal of contaminated rRNA with RiboMinus transcriptome isolation kit (Invitrogen). Total RNA samples used for RT-PCR were isolated by using RNeasy kit (Qiagen) with an additional DNase-I digestion step on column.

### LC-MS/MS

A unit of 200–300 ng of mRNA was digested by nuclease P1 (1U) (Wako) in 26 μL of buffer containing 20 mM of CH_3_COONH_4_ (pH 5.3) at 42 °C for 2 h, followed by additions of NH_4_HCO_3_ (1 M, 3 μL) and alkaline phosphatase (1 μL, 1 U/μL; Sigma) and incubation at 37 °C for 2 h. The sample was diluted to 50–60 μL and then filtered (0.22-μm pore size, 4 mm diameter, Millipore), and 10 μL of the solution was injected into LC-MS/MS. The nucleosides were separated by reverse-phase ultra-performance liquid chromatography on a C18 column with online mass spectrometry detection using Agilent 6410 QQQ triple-quadrupole LC mass spectrometer in positive electrospray ionization mode. The nucleosides were quantified by using the nucleoside to base ion mass transitions of 282 to 150 (m^6^A), and 268 to 136 (A). Quantification was performed in comparison with the standard curve obtained from pure nucleoside standards running on the same batch of samples. The ratio of m^6^A to A was calculated based on the calibration curves.

### Purification of METTL3, METTL14, WTAP, and VIRMA protein interactomes and mass spectrometry identification

The previously published protocol was followed^28^. Briefly, stable expression HeLa cell lines with dual-tagged METTL3, METTL14, and WTAP (N-terminal Flag and HA in tandom) were created by puromycin selection. The control cell line with expression of only tandem Flag and HA peptides was created similarly. Thirty 15-cm plates of METTL3, METTL14, WTAP, or control HeLa stable line cells were collected by cell lifter and suspended in ice-cold phosphate-buffered saline (PBS; 5 mL/plate). The cell pellets were pooled and washed once with 30 mL cold PBS. A volume of 30 mL hypotonic buffer (10 mM Tris, pH 7.4, 10 mM KCl, and 1.5 mM MgCl_2_) was added into the tube. The pellets were resuspended by inverting the tube and swollen after 15 min incubation on ice. The cells were collected at 3000 r.p.m. for 10 min at 4 °C. Equal volume of hypotonic buffer to pellet with protease inhibitor and SUPERasin was added. The mixture was homogenizd by Douncing pestle 25–30 times and then was spun down at 3500 r.p.m. at 4 °C for 15 min. The resultant supernatant (cytoplasmic part) was discarded and the pellet (nuclear part) was resuspended with half volume of low salt buffer (20 mM Tris, pH 7.4, 25 vol% glycerol, 1.5 mM MgCl_2_, 0.2 mM EDTA, pH 8, and 20 mM KCl) with protease inhibitor and SUPERasin. The mixture was homogenized five times and poured into a small beaker. Calculated volume of high-salt buffer (20 mM Tris, pH 7.4, 25 vol% glycerol, 1.5 mM MgCl_2_, 0.2 mM EDTA, pH 8, and 1.2 M KCl) with protease inhibitor and SUPERasin was added dropwise by syringe under stirring to adjust final KCl concentration around 400 mM in the mixture. After dropping, stir for another 30 min and centrifuge at 15 000 r.p.m. for 30 min at 4 °C. The supernatant was dialyzed against 1 L BC-100 (20 mM Tris, pH 7.4, 100 mM KCl, 20% glycerol, 0.2 mM EDTA, pH 8, and 0.5 mM dithiothreitol (DTT)) with protease inhibitor and SUPERasin for 3 h at 4 °C and overnight for second dialysis. The dialyzed nuclear extract was spun down at 15 000 r.p.m. for 30 min at 4 °C and could be stored at −80 °C for further experiments. The pellet was dissolved in the appropriate volume of medium-salt buffer (20 mM Tris, pH 7.4, 400 mM KCl, 20% glycerol, 0.2 mM EDTA, pH 8, and 0.5 mM DTT) with protease inhibitor and SUPERasin, and centrifuged again at 10 000 r.p.m. for 30 min at 4 °C. The supernant was transferred and saved in a new tube.

A volume of 400 μL anti-Flag magnetic beads (Sigma) were washed by washing buffer (50 mM Tris, pH 7.9, 100 mM KCl, 5 mM MgCl_2_, 0.2 mM EDTA, 0.5 mM DTT, 0.1% NP-40, 10% glycerol, protease inhibitor, and SUPERasin) three times, combined with the nuclear extract, and incubated at 4 °C for 4 h with rotation. The beads were then washed by washing buffer (without SUPERasin) four times, followed by incubation with 500 μL elution solution containing 3× Flag peptide (0.3 mg/mL in washing buffer, Sigma) at 4 °C for 1 h. During this time, 50 μL anti-HA magnetic beads (Pierce) were washed three times with washing buffer. The eluted samples were incubated with anti-HA beads for 3 h at 4 °C followed by four washes with washing buffer (without SUPERasin). The final protein complex was eluted by 300 μL elution buffer (0.3 mg/mL HA peptide in washing buffer, Sigma), purified by trichloroacetic acid precipitation and trypsin digestion. The protein mass spectroscopy was performed by the Institutes of Biomedical Sciences at Fudan University, Shanghai.

A similar procedure was applied to identify VIRMA interactome. The VIRMA construct in pTriEx 1.1-Neo with only Flag tag was overexpressed in HeLa cells and Flag IP was used to purify VIRMA interactome.

### Protein co-IP and western blotting validation

Stable line cells expressing Flag- and HA-tagged METTL3, METTL14, and WTAP, and Flag-HA peptide itself (control), and cells overexpressing different forms of VIRMA were collected by cell lifter (three 15-cm plates for each), and pelleted by centrifuge at 1000 r.p.m. for 5 min. The cell pellets were resuspended with two volumes of lysis buffer (150 mM KCl, 10 mM HEPES, pH 7.6, 2 mM EDTA, 0.5% NP-40, 0.5 mM DTT, protease inhibitor cocktail, and RNase inhibitor), and incubated on ice for 10 min. To remove the cell debris, the lysate solution was centrifuged at 17 000 r.p.m. for 15 min at 4 °C. The resulting supernatant was further cleared by passing through a 0.45-μm membrane syringe filter. While 50 μL of cell lysate was saved as input, the rest was incubated with the anti-Flag M2 magnetic beads (Sigma) in ice-cold NT2 buffer (200 mM NaCl, 50 mM HEPES, pH 7.6, 2 mM EDTA, 0.05% NP-40, 0.5 mM DTT, and RNase inhibitor) for 4 h at 4 °C. Afterwards, the beads was subject to extensive wash with 8 × 1 mL portions of ice-cold NT2 buffer, followed by incubation with the elution solution containing 3× Flag peptide (0.3 mg/mL in NT2 buffer, Sigma) at 4 °C for 2 h or direct incubation with SDS loading dye. The eluted samples, saved as IP, were analyzed by western blotting.

### Expression and purification of VIRMA and ZC3H13

Full-length human *VIRMA* and *ZC3H13* (obtained from HeLa cDNA) were subcloned into a modified version of pTriEx-1.1-Neo (with a strong translational enhancer sequence SP163 added right before the start codon ATG and a 3× Flag tag at C terminus). The plasmids were transfected into HEK 293T cells at the confluency of 80–90% with PEI (Polysciences, high potency linear, *M*_w_ = 40 000). Cells were harvested with cell lifter 24 h after transfection, pelleted by centrifuge at 2000 r.p.m. at 4 °C for 5 min, and washed once with cold PBS. The cell pellets were resuspended with two to three packed cell volume of lysis buffer (150 mM KCl, 10 mM HEPES, pH 7.6, 2 mM EDTA, 0.5% NP-40, 0.5 mM DTT, and 1/100 protease inhibitor cocktail), pipetted up and down several times, incubated on ice for 15 min, treated with ultrasonic for 2 min, and then centrifuged at 14 000 r.p.m. for 15 min at 4 °C, the supernatant was passed through a 0.22-μm membrane syringe filter and saved 50 μL as input. The rest was incubated with anti-flag M2 affinity gel in ice-cold NT2 buffer (200 mM NaCl, 50 mM HEPES, pH 7.6, 2 mM EDTA, 0.05% NP-40, 0.5 mM DTT, and 1/100 protease inhibitor cocktail) for 4 h at 4 °C. Then, the gel was washed with ice-cold NT2 buffer four times and centrifuged at 8000 × *g* for 3 min to collect the gel. Afterwards, 60 μL 2× SDS loading dye was added, and the whole mixture was boiled at 94 °C for 5 min and centrifuged at 8000 × *g* for 3 min. The supernatant was saved as IP. The IP products were then analyzed by SDS-polyacrylamide gel electrophoresis using Colloidal Coomassie staining with G-250 (0.02% (w/v) G-250, 5% (w/v) aluminum sulfate-(14–18)-hydrate, 10% (v/v) ethanol, and 2% (v/v) orthophosphoric acid in ultrapure water)^40^.

### WTAP rescue assay

The siRNAs for control and *VIRMA* were transfected into HeLa cells at the confluency of 20–30% (two *VIRMA* siRNA sequences used). For cells treated with siVIRMA, control plasmid, and WTAP plasmid were transfected into HeLa cells 48 h after siRNA transfection. Cells were harvested after another 24 h. About 1/8 cells were used for western blotting validation, and the rest cells were treated with Trizol for total RNA extraction. Total RNAs were subjected to two rounds of poly(A) selection to obtain polyadenylated RNAs. For each sample, about 300 ng of mRNA was used for LC-MS/MS to quantify m^6^A level.

### Cell proliferation assay

A total of 5000 cells were seeded per well in a 96-well plate. The effect of *VIRMA* depletion on cell proliferation was assessed in HeLa cells by assaying cells at various time points using the CellTiter 96 Aqueous One Solution Cell Proliferation Assay (Promega) following the manufacturer’s protocols. For each cell line tested, the signal from the MTS assay was normalized to the value observed ~24 h after seeding. MTS is [3-(4,5-dimethylthiazol-2-yl)-5-(3-carboxymethoxyphenyl)-2-(4-sulfophenyl)-2H-tetrazolium.

### RNA stability assay

Two 10-cm plates of HeLa control and *VIRMA*^mut/−^ cells at 80% confluency were re-seeded into three 6-cm plates separately, and each plate was controlled to contain the same amount of cells. After 48 h, actinomycin D (Sigma, A9415) was added to 5 µg/mL at 6, 3, and 0 h before trypsinization collection. The total RNA was purified by RNeasy kit with an additional DNase-I digestion step on column. The degradation rate of RNA (*k*) was estimated by the following equation

$$\ln \left( {\frac{{N_t}}{{N_0}}} \right){\mathrm{ = }} - kt$$

where *t* is the TI time (h), *N*_*t*_ and *N*_0_ stand for the RNA quantities at time *t* and 0. Through exponential decay fitting of *N*_*t*_/*N*_0_ versus time, the *k* can be derived and thus the half-life (*t*_1/2_) can be calculated.

$$t_{\frac{1}{2}}{\mathrm{ = }}\frac{{{\mathrm{ln}}2}}{k}$$

### m^6^A-seq

Total RNA was isolated from HeLa control and *VIRMA*^mut/-^ cells with TRIZOL reagent. Polyadenylated RNA was further enriched from total RNA by using FastTrack MAGMaxi mRNA isolation kit (Invitrogen). In particular, an additional DNase-I digestion step was applied to all the samples in order to avoid DNA contamination. RNA fragmentation, m^6^A-seq, and library preparation were performed according to the previously published protocol^33^. Each experiment was conducted in two biological replicates.

### m^6^A-seq data analysis

m^6^A-seq data were analyzed according to the protocols described before^33, 41^. Significant peaks with FDR < 0.05 were annotated to RefSeq database (hg19). Sequence motifs were identified by using Homer. Gene expression was calculated by Cufflinks using the sequencing reads from input samples. Cuffdiff was used to find the DE genes.

### VIRMA RIP-seq

Ten 15-cm plates of HeLa cells were collected by cell lifter, pelleted by centrifuge at 2000 r.p.m. at 4 °C for 5 min, and washed with cold PBS once. The cell pellet was resuspended with three packed cell volume of lysis buffer (150 mM KCl, 2 mM EDTA, 0.5% NP-40, 0.5 mM DTT, 50 mM HEPES, pH 7.5, 1/100 protease inhibitor cocktail, 200 U/mL RNase inhibitor; one plate with about 150 μL cell pellet and 450 μL lysis buffer), pipetted up and down several times and incubated on ice for 15 min, and treated with ultrasonic for 1 min. After centrifugation at 14 000 r.p.m. for 30 min at 4 °C, the supernatant was passed through a 0.22-μm membrane syringe filter, saved 1/50 as input and mixed with 1 mL Trizol for RNA extraction and followed by two rounds of poly(A) selection to get mRNA, saved as input mRNA. The remaining cell lysate was used for endogenous VIRMA IP as follows: the cell lysate was incubated with 20 μg anti-VIRMA rabbit polyclonal antibody (Bethyl, A302-124A-M) at 4 °C overnight, 200 μL protein A beads were thrice washed with binding buffer (300 mM KCl, 1.5 mM MgCl_2_, 0.05% NP-40, 2 mM EDTA, 0.5 mM DTT, and 50 mM HEPES, pH 7.5), resuspended in 500 μL binding buffer (1/100 protease inhibitor cocktail and 200 U/mL RNase inhibitor added), and incubated with cell lysate-antibody mixture at 4 °C for another 4 h. The protein A beads were collected with magnetic stand, thrice washed with binding buffer (1/100 protease inhibitor cocktail and 200 U/mL RNase inhibitor added), and then mixed with 500 μL Trizol to get RNA and saved as IP. The libraries for both input mRNA and IP were constructed following Illumina′s standard protocol.

### RIP data analysis

Sequencing reads of RIP samples were mapped to hg19 reference genome using Hisat software, with multi-mapping reads excluded. Cufflinks was used to normalize mapped samples by geometric normalization method. RIP-seq-enriched genes were defined as FDR ≤ 0.05 and log_2_(IP/input) ≥ 1.

### Tethering assay

The three forms of VIRMA tagged with a lambda peptide at C terminus were subcloned into pTriEx 1.1-Neo (Novagen) using restriction sites of *Nco*I and *Age*I. The control plasmid contains lambda peptide only. The reporter plasmid (pmirGLO-dual luciferase-5BoxB) and effecter plasmids (λ, VIRMA-λ, N-VIRMA-λ, and C-VIRMA-λ in pTriEx 1.1-Neo) were transfected into HeLa cells at ratio of 1:9 at about 80% confluency (each for one 10-cm plate). The transfection mixture was changed with fresh medium after 6 h and the cells transfected with different versions of VIRMA and control plasmids were harvested after 24 h with Trizol Reagent for total RNA and all subjected to two rounds of poly(A) selection for mRNA purification. About 500 ng of mRNA was saved as input, the rest mRNA (about 3 μg) together with 6 μL (3 μg) m^6^A-antibody and 20 μL protein A beads (washed with IP buffer at least thrice before use) were incubated with 200 μL m6A-IP buffer (50 mM Tris-HCl, 750 mM NaCl, and 0.5% (vol/vol) Igepal CA-630) in the presence of RNasin for 6 h, the supernatant was saved as FT. Protein A beads binding m^6^A-containing mRNA were washed with IP buffer in the presence of RNasin for three to five times and mixed with 300 μL Trizol reagent for RNA extraction and saved as IP. Equal amount of input, IP, and FT were used for qRT-PCR to detect the relative mRNA level of Firefly luciferase in input, IP,and FT, and also mRNA level of GAPDH was detected in input and FT as internal control and then calculated for relative m^6^A methylation level.

### Analysis of expression from RNA-seq

Sequencing reads were mapped to the hg19 reference genome using HISAT software. After multi-mapped reads were removed, FPKM values were calculated with Cuffnorm software.

### Analysis of APA from RNA-seq

DaPars was used for the de novo identification of APA from RNA-Seq data^42^. FDR cutoff for DaPars was 0.05, the absolute difference cutoff of mean PDUIs was set to 0.2, and the absolute log_2_ ratio (fold change) cutoff of mean PDUIs must be no <1. In order to avoid false positive estimation on low-coverage transcripts, 30-fold coverage cutoff on the 3′UTR region of all samples was also set. Pair-end 150 bp sequencing mode were applied for APA analysis.

### Distal polyadenylation site usage assay

qRT-PCR was performed to investigate distal polyadenylation site (dPAS) usage of selected genes in CPSF5 knockdown cells, including *SPEN*, *NOTCH1*, *TACC2*, and *PPP1R13B*, whose 3′UTRs were found shortened after CPSF5 knockdown through RNA-seq. The siRNA targeting CPSF5 and a control siRNA were transfected into HeLa cells, respectively, at the confluency of 20–30% using LipoRNAiMax and cells were harvested 48 h later. Total RNA was extracted with Trizol Reagent following the manufacturer’s protocol, cDNA was generated using Primescript First Strand cDNA synthesis kit from Takara and the qRT-PCR reactions were performed with Bio-Rad CFX96 system. The common primers were designed to target the CDSs and distal primers were designed to target sequences just before the dPASs. The relative dPAS usage was calculated using the 2^−ΔΔΔCT^ method^38^, where CT is the cycle number to reach the detection threshold. First of all, the CT values of common and distal amplicons were normalized with GAPDH, where ΔCT (common or distal) = CT_common or distal_–CT_GAPDH_, ΔΔCT = ΔCT_dstal_–ΔCT_common_, and ΔΔΔCT = ΔΔCT_target siRNA_–ΔΔCT_control siRNA_, then the formula 2^−ΔΔΔCT^ was utilized in order to measure the dPAS usage of target genes in CPSF5 knockdown cells relative to control cells.

### Accession codes

The high-throughput sequencing data reported in this paper has been deposited in Gene Expression Omnibus database, www.ncbi.nlm.nih.gov/geo (accession no. GSE102493).

## Electronic supplementary material

Table S1

Table S2

Table S3

Table S4

Table S5

Table S6

Table S7

Table S8

Table S9

Table S10

Supplementary information
