# Supplementary material for: VIRMA mediates preferential m6A mRNA methylation in 3′UTR and near stop codon and associates with alternative polyadenylation
Source: Cell Discov. 2018 Feb 27;4:10. doi: 10.1038/s41421-018-0019-0 (PMC5826926; doi:10.1038/s41421-018-0019-0)
Supplement: Supplementary file 2 — Table S2 [file 41421_2018_19_MOESM2_ESM.pdf]

Supplementary Table S2 Brief summary of high throughput sequencing data[a]

No.	Samples	Raw reads	Mappable reads	Mappableratio[b]
1	Ctrl-Input1	55,597,529	47,313,497	0.851
2	Ctrl-Input2	64,072,102	53,692,421	0.838
3	Ctrl-m^{6}A-IP1	24,143,793	19,942,773	0.826
4	Ctrl-m^{6}A-IP2	57,519,817	47,051,210	0.818
5	VIRMA-Mut-Input1	48,195,956	40,629,190	0.843
6	VIRMA-Mut-Input2	96,802,916	80,443,223	0.831
7	VIRMA-Mut-m^{6}A-IP1	24,471,921	19,161,514	0.783
8	VIRMA-Mut-m^{6}A-IP2	74,883,968	58,035,075	0.775
9	Ctrl-PE-Input1	105,409,978	90,847,257	0.862
10	Ctrl-PE-Input2	92,685,858	77,196,032	0.833
11	Ctrl-PE-m^{6}A-IP1	160,231,915	137,661,993	0.859
12	Ctrl-PE-m^{6}A-IP2	118,647,121	98,561,493	0.831
13	VIRMA-Mut-PE-Input1	167,345,956	153,763,315	0.919
14	VIRMA-Mut-PE-Input2	100,602,262	93,748,630	0.932
15	VIRMA-Mut-PE-m^{6}A-IP1	104,734,545	97,665,586	0.933
16	VIRMA-Mut-PE-m^{6}A-IP2	110,636,259	102,603,591	0.927
17	VIRMA-RIP Input1[c]	95,885,008	75,269,731	0.785
18	VIRMA-RIP Input2[c]	75,525,136	60,269,058	0.798
19	VIRMA-RIP-IP1[c]	65,691,212	20,758,422	0.316
20	VIRMA-RIP-IP2[c]	53,309,821	21,217,308	0.398
21	Wild-Ctrl-Input1[c]	87,982,606	75,401,093	0.857
22	Wild-Ctrl-Input2[c]	55,543,410	44,767,988	0.806
23	Wild-Ctrl-m^{6}A-IP1[c]	79,509,346	68,775,584	0.865
24	Wild-Ctrl-m^{6}A-IP2[c]	87,863,226	73,365,793	0.835
25	METTL3-Mut-Input1	113,320,039	103,914,475	0.917
26	METTL3-Mut-Input2	47,168,770	43,772,618	0.928
27	METTL3-Mut-IP1	51,338,209	43,380,786	0.845
28	METTL3-Mut-IP2	69,046,091	58,620,131	0.849
29	siCtrl-1	62,405,838	48,676,553	0.780
30	siCtrl-2	78,398,888	61,856,722	0.789
31	siCtrl-m^{6}A-IP1	52,278,909	45,587,208	0.872
32	siCtrl-m^{6}A-IP2	59,604,940	50,664,199	0.850
33	siCPSF5-1[d]	60,298,336	45,284,050	0.751
34	siCPSF5-2[d]	229,749,097	182,191,034	0.793
35	siCPSF5-m^{6}A-IP1[d]	49,315,231	37,430,260	0.759
36	siCPSF5-m^{6}A-IP2[d]	65,009,905	54,933,369	0.845
37	siZC3H13-1[e]	91,340,429	72,432,960	0.793
38	siZC3H13-2[e]	32,231,869	21,885,439	0.679
39	siZC3H13-m^{6}A-IP1[e]	90,635,657	73,686,789	0.813

40	siZC3H13-m^{6}A-IP2[e]	124,874,423	102,022,404	0.817
41	siCtrl-for HAKAI-1	72,648,322	60,370,755	0.831
42	siCtrl-for HAKAI-2	86,436,500	72,001,604	0.833
43	siCtrl-for HAKAI-m^{6}A-IP1	60,841,492	51,471,902	0.846
44	siCtrl-for HAKAI-m^{6}A-IP2	121,318,427	105,061,758	0.866
45	siHAKAI-1[f]	46,096,632	39,781,393	0.863
46	siHAKAI-2[f]	54,773,938	47,050,812	0.859
47	siHAKAI-m^{6}A-IP1[f]	64,197,384	54,375,184	0.874
48	siHAKAI-m^{6}A-IP2[f]	85,650,847	75,115,792	0.877
49	siCtrl-for VIRMA-1	54,197,474	49,345,604	0.910
50	siCtrl-for VIRMA-2	30,221,930	27,547,503	0.912
51	siCtrl-for VIRMA-m^{6}A-IP1	47,367,315	41,308,384	0.872
52	siCtrl-for VIRMA-m^{6}A-IP2	46,462,849	40,549,305	0.873
53	siVIRMA-1[g]	42,409,759	38,697,076	0.912
54	siVIRMA-2[g]	45,903,034	42,066,729	0.916
55	siVIRMA-m^{6}A-IP1[g]	48,759,125	44,219,723	0.907
56	siVIRMA-m^{6}A-IP2[g]	48,877,923	44,400,333	0.908

[a]Sequencing was performed using single end of 50 bp for samples 1–8 and pair end of 150 bp for rest samples. Samples 1–8, 9–16, 17-28, 29–48, and 49–56 were prepared and sequenced in the same batches, respectively. Two biological replicates for each experimental condition in HeLa cell. The same siRNA control was used for CPSF5 and ZC3H13 knockdowns. Ctrl = Control; VIRMA-Mut = *VIRMA*$^{mut/-}$ construct 1; METTL3-Mut = *METTL3*$^{mut/-}$; PE = pair end of 150 bp sequencing, IP = immunoprecipitation; RIP = RNA immunoprecipitation. [b]Mappable ratio = Mappable reads/Raw reads. [c]The wild-type control m^{6}A-seq data and VIRMA RIP-seq data was obtained in the same batch and was used for comparison in bioinformatic analysis. [d]The siRNA named siCPSF5-1 in Supplementary Table 9 was used. [e]The siRNA named siZC3H13-1 in Supplementary Table 9 was used. [f]The siRNA named siHAKAI-1 in Supplementary Table 9 was used. [g]The siRNA named siVIRMA-1 in Supplementary Table 9 was used.
