# Supplementary material for: VIRMA mediates preferential m6A mRNA methylation in 3′UTR and near stop codon and associates with alternative polyadenylation
Source: Cell Discov. 2018 Feb 27;4:10. doi: 10.1038/s41421-018-0019-0 (PMC5826926; doi:10.1038/s41421-018-0019-0)
Supplement: Supplementary file 11 — Supplementary information [file 41421_2018_19_MOESM11_ESM.pdf]

Supplementary information

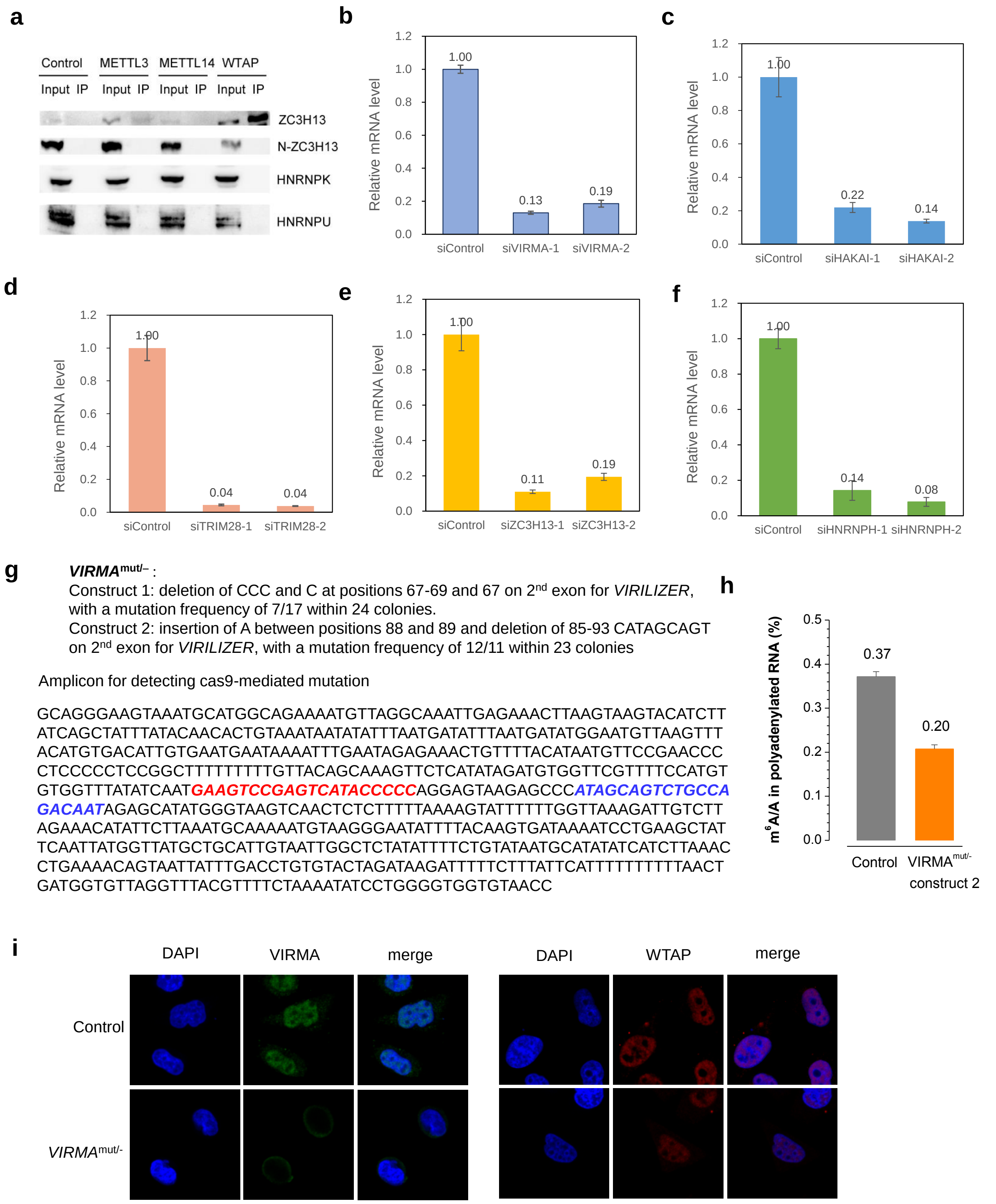

Supplementary Figure S1 (**a**) Validation of selected common targets, ZC3H13, HNRNPK and HNRNPU by western blotting. Flag-tagged full length ZC3H13 and N-terminal ZC3H13 (N-ZC3H13) were tested using Flag antibody after HA IP of METTL3, METTL14, and WTAP stable cell lines. (**b-f**) The qPCR results of siRNA knockdowns of *VIRMA*, *HAKAI*, *TRIM28*, *ZC3H13*, and *HNRNPH*, respectively, by using two independent siRNA sequences.

P values calculated using one-tailed Student's *t*-test between control and knockdown samples were less than 0.05. (**g**) CRISPR-cas9 was used to generate the *VIRMA*$^{mut/-}$ cell lines. The sequence in red and blue are the sgRNA-targeted regions for constructs 1 and 2. The PCR-amplified genomic sequence containing the sgRNA-targeted region is shown. The gene-editing result was derived from TA-cloning. (**h**) Comparison of m^6A levels in polyadenylated nuclear RNAs in between control and *VIRMA*$^{mut/-}$ construct-2 HeLa cell lines. Data is represented as means ± SEM of $n = 3$. *P* values calculated using one-tailed Student's *t*-test between control and *VIRMA*$^{mut/-}$ samples are less than 0.001. (**i**) Immunostains of VIRMA and WTAP were compared between *VIRMA*$^{mut/-}$ and control cell lines.

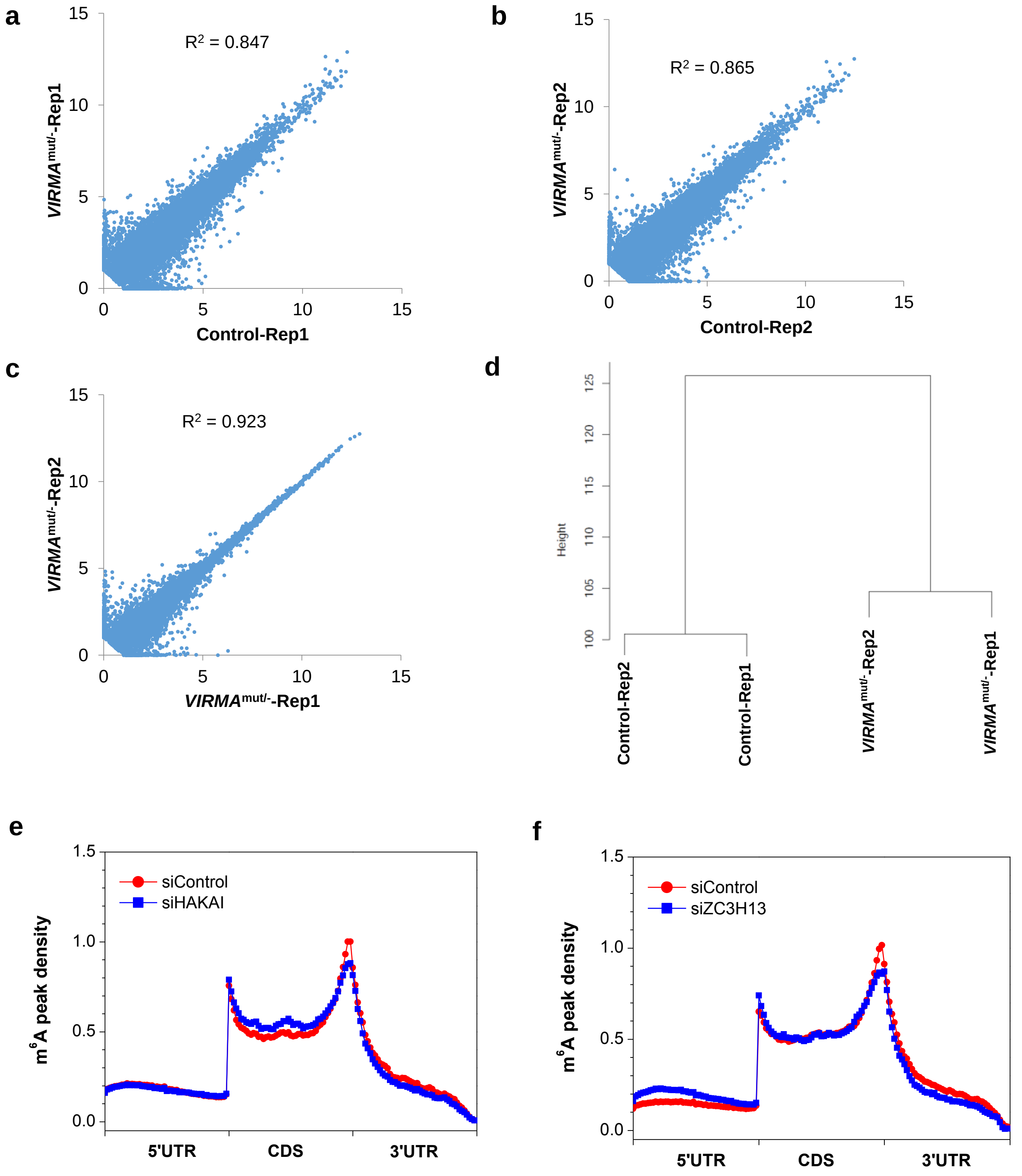

Supplementary Figure S2 (**a**, **b**) RNA-seq analysis for control and *VIRMA*$^{mut/-}$cell lines (2 replicates for each). Scatter plots for the expression comparison between control and *VIRMA*$^{mut/-}$. Each dot represents one gene with expression FPKM value calculated by Cufflinks. The Fig. indicates R-squared value in order to show the relationship between two series of data. (**c**) Scatter plot for expression comparison between two *VIRMA*$^{mut/-}$ replicates. (**d**) Clustering analysis for 2 control samples and 2 *VIRMA*$^{mut/-}$ samples. (**e**, **f**) Effects of depletion of *HAKAI* (**e**) and *ZC3H13* (**f**) by siRNA knockdown on the profiles of m^{6}A peak density along mRNA transcript.

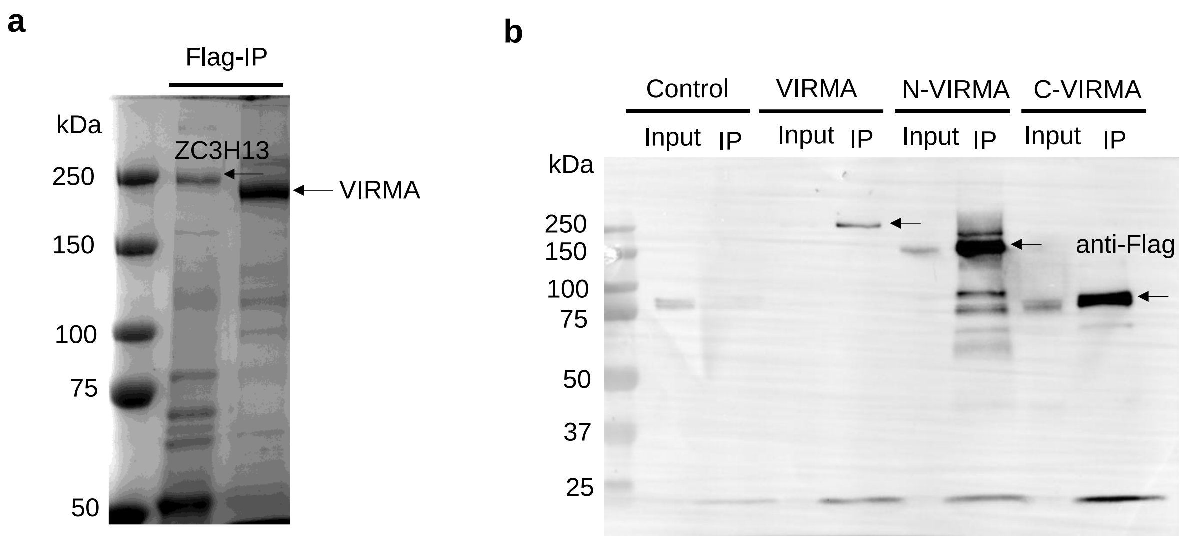

Supplementary Figure S3 (**a**) Commassie blue staining image of PAGE gel of IP products of Flag-tagged VIRMA and ZC3H13 overexpressed in HEK 293FT cells. (**b**) Western blots of IP products of Flag-tagged VIRMA and its truncated forms, including N-term and C-term. All these constructs were overexpressed in HeLa cells.

a

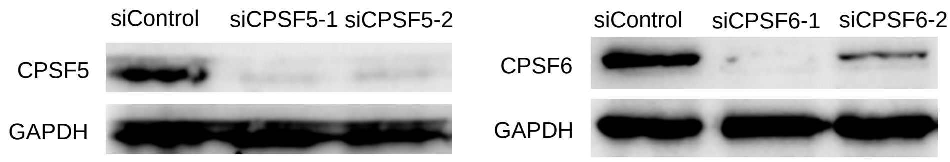

b

***METTL3*$^{mut/-}$** : deletion of CT and TGC at positions 125-126 and 123-125 of 3rd exon for *METTL3* with a mutation frequency of 22/8 within 30 colonies

Amplicon for detecting cas9-mediated mutation

TCCACGGTAATGACCTGACAGAGTTAGGATTTACCACTTATTTTCAGTTCACGGTCAGTGTCTTATTGCCCATACCT
CCCCTCGTTCTCTAGCCAGATGCTCCTGCCACTCAAGATGGGGTAGAAAGCCTCCTGCAGAAGTTTGCAGCTCAG
GAGTTGATTGAGGTAAAGCGAGGTCTCCTACAAGATGATGCACATCCTACTCTTGTAACCT***ATGCTGACCATTCCA***
AGCTCTCTGCCATGATGGGTGCTGTGGCAGAAAAGAAGGGCCCTGGGGAGGTAGCAGGGACTGTCACAGGGCA
GAAGCGGCGTGCAGAACAGGACTCGACTACAGTAGCTGCCTTTGCCAGTTCGTTAGTCTCTGGTCTGAACTCTTC
AGCATCGGAACCAGCAAAGGAGCCAGCCAAGAAATCAAGGAAACATGCTGCCTCAGATGTTGATC

c

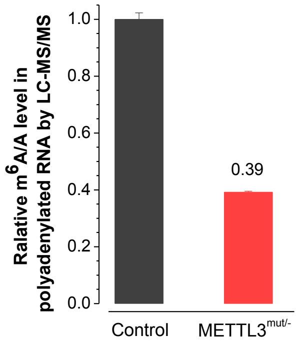

d

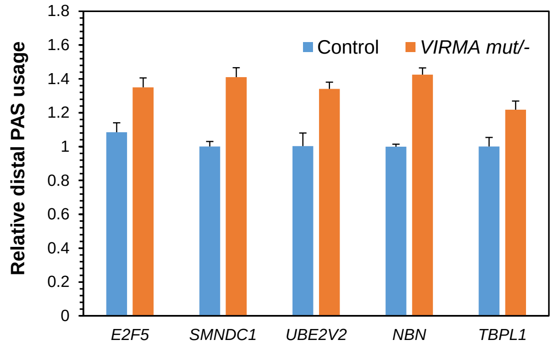

e

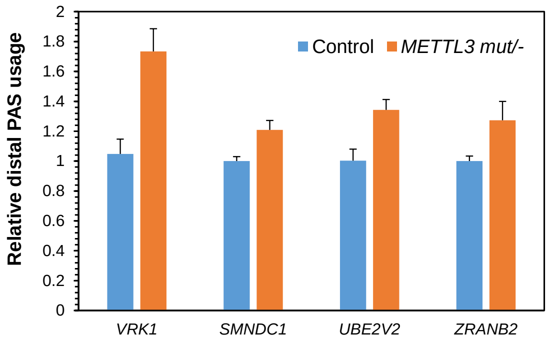

f

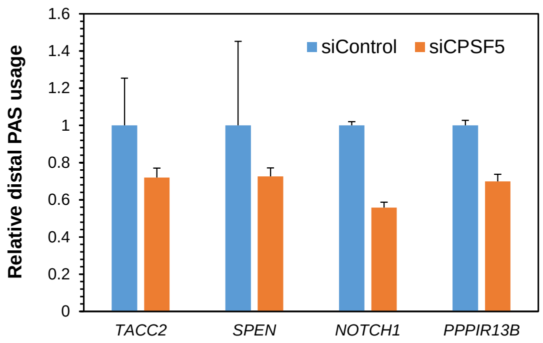

g

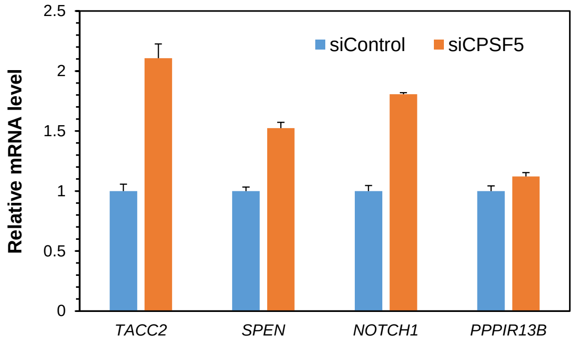

Supplementary Figure S4 (**a**) Western blots showing siRNA knockdowns of CPSF5 and CPSF6, respectively, by using two independent siRNA sequences. GAPDH was used as an internal control. (**b**)The *METTL3*$^{mut/-}$ cell line was generated by CRISPR-cas9.The sequence in italic and red is the sgRNA-targeted region. The PCR-amplified genomic sequence containing the sgRNA-targeted region is shown. The gene-editing result was derived from TA-cloning. (**c**) Comparison of mRNA m^{6}A level in between control and*METTL3*$^{mut/-}$ cell lines. The m^{6}A level was measured using UHPLC-QQQ-MS/MS. *P* values calculated using one-tailed Student's *t*-test between control and *METTL3*$^{mut/-}$samples are less than 0.001. Data are represented as means ± SEM of *n* = 3. (**d**, **e**) Validation of selected targets with 3`UTR lengthening in *VIRMA*$^{mut/-}$ and *METTL3*$^{mut/-}$ versus control cells. *P* values calculated using one-tailed Student's *t*-test between control and mutants samples are less than 0.05. Data are represented as means ± SEM of *n* = 3. (**f, g**) Comparison of distal PAS usage and mRNA level of selected m^{6}A-containing genes under siControl and siCPSF5 quantified by qPCR. *P* values calculated using one-tailed Student's *t*-test between control and mutants samples are less than 0.05. Data are represented as means ± SEM of *n* = 3.

Human *VIRMA* full length (KIAA1429 Isoform 1, NM_015496.4)

Nucleotide sequence: 5439 nt (Sequence in red is defined as *VIRMA* C-terminal)
ATGGCGGTGGACTCGGCGATGGAGCTGTTATTTTTAGATACTTTTAAACACCCGAG
CGCTGAGCAAAGTTCTCATATAGATGTGGTTCGTTTTCCATGTGTGGTTTATATCAA
TGAAGTCCGAGTCATACCCCCAGGAGTAAGAGCCCATAGCAGTCTGCCAGACAAT
AGAGCATATGGAGAGACATCTCCCCATACATTTCAATTAGACTTATTCTTCAACAAT
GTAAGCAAACCAAGTGCCCCTGTTTTCGATAGGTTGGGAAGCCTGGAATATGATG
AGAATACTTCCATCATCTTTAGACCTAACTCAAAGGTGAATACTGATGGTCTGGTG
CTAAGAGGCTGGTATAACTGTCTGACACTGGCAATATATGGATCAGTGGATAGAGT
GATAAGTCATGACAGAGACTCTCCACCACCACCTCCACCGCCACCACCTCCC
CAGCCACAACCAAGTTTGAAAAGGAATCCAAAACATGCTGATGGGGAGAAAGAA
GATCAGTTTAATGGAAGCCCTCCAAGACCACAGCCAAGGGGACCAAGAACTCCTC
CAGGACCCCCTCCACCTGATGATGATGAAGATGATCCTGTGCCTCTGCCAGTGTCT
GGTGACAAGGAAGAGGATGCTCCTCATAGAGAAGATTACTTTGAGCCCATTTCTC
CTGATCGGAATTCTGTTCCCCAGGAAGGGCAATATTCTGATGAAGGAGAAGTAGA
AGAGGAACAACAAGAAGAAGGAGAAGAAGATGAAGATGATGTGGATGTAGAGG
AAGAAGAGGATGAGGATGAGGATGATCGACGAACAGTAGACAGTATTCCTGAGG
AGGAAGAGGAAGATGAAGAGGAAGAAGGTGAAGAGGATGAAGAAGGTGAAGGG
GATGATGGTTATGAACAAATTTCCAGTGATGAAGATGGAATTGCTGACTTGGAACG
TGAAACATTTAAGTATCCAAACTTTGATGTTGAATACACTGCTGAAGACTTAGCTT
CAGTTCCTCCTATGACATATGATCCATATGACAGGGAGCTTGTACCACTCTTATACT
TCAGTTGTCCATACAAGACTACTTTTGAAATTGAAATCAGTAGAATGAAGGATCAA
GGTCCAGATAAAGAAAATTCAGGGGCAATCGAAGCCTCAGTGAAGTTAACAGAA
CTCTTAGATTTGTATAGAGAAGATAGAGGTGCAAAATGGGTAACAGCTTTAGAAGA
AATTCCAAGTTTAATAATAAAAGGGTTAAGCTATTTGCAATTGAAAAACACAAAAC
AAGACTCCCTTGGCCAGTTGGTAGACTGGACCATGCAAGCTTTAAATTTACAAGTA

GCGCTTCGCCAACCTATCGCCTTAAATGTTCGACAGCTCAAAGCTGGGACCAAATT
AGTGTCCTCACTAGCAGAATGTGGGGCTCAAGGAGTTACAGGACTGCTACAAGCA
GGAGTGATCAGTGGATTATTTGAACTTCTGTTTGCTGATCACGTATCATCTTCTCTT
AAGTTAAATGCTTTTAAAGCTTTGGACAGTGTCATTAGTATGACAGAAGGAATGGA
AGCTTTTTTAAGAGGTAGGCAGAATGAAAAAAGTGGTTATCAAAAGCTTCTGGAA
CTCATACTTTTAGATCAGACTGTGAGGGTTGTTACTGCTGGTTCAGCTATTCTCCAA
AAATGCCATTTCTATGAAGTCTTGTCAGAGATTAAAAGACTTGGTGACCATTTAGC
AGAGAAGACTTCATCTCTTCCTAACCACAGTGAACCTGATCACGACACAGATGCT
GGACTTGAGAGAACAAACCCAGAATATGAAAATGAGGTGGAAGCTTCTATGGATA
TGGATCTTTTGGAATCCTCAAATATAAGTGAAGGGGAAATAGAAAGGCTTATTAAC
CTCCTAGAAGAAGTTTTTCATTTAATGGAAACTGCCCCTCATACAATGATCCAACA
ACCTGTTAAGTCTTTCCCAACGATGGCACGAATTACTGGACCTCCAGAGAGGGAT
GATCCATACCCTGTTCTCTTTAGATATCTTCACAGTCATCACTTCTTGGAGTTGGTT
ACCTTGCTTCTGTCAATTCCAGTAACAAGTGCTCACCCTGGTGTGCTGCAAGCCAC
AAAAGATGTTTTGAAGTTTCTTGCACAGTCACAGAAGGGTCTTCTTTTTTTTATGT
CGGAATATGAAGCAACAAATTTATTGATCCGAGCTCTGTGTCACTTTTATGATCAAG
ATGAGGAGGAAGGTCTCCAATCTGATGGTGTTATTGATGATGCATTTGCCTTGTGG
CTACAGGACTCAACACAGACATTGCAATGTATTACAGAACTGTTCAGCCATTTTCA
GCGTTGTACAGCCAGTGAAGAAACAGACCATTCAGATCTCTTGGGAACCCTGCAC
AATCTTTATTTGATTACTTTTAATCCTGTGGGAAGATCAGCTGTTGGCCATGTTTTTA
GTCTGGAGAAAAATCTCCAAAGTCTTATTACTCTAATGGAGTACTATTCCAAAGAA
GCCTTGGGTGATTCCAAATCTAAGAAGTCAGTAGCTTATAATTACGCATGCATACTT
ATTTTGGTGGTGGTTCAGTCTTCCAGTGATGTTCAAATGCTAGAACAACATGCAGC
ATCTCTCTTGAAGCTTTGTAAAGCAGATGAAAATAATGCTAAATTGCAAGAACTTG
GCAAGTGGCTTGAACCTCTGAAAAACCTTAGATTTGAAATTAACTGCATCCCAAA
CTTAATTGAGTATGTTAAGCAGAATATCGATAACTTGATGACCCCAGAAGGAGTTG
GCCTTACCACTGCCTTACGTGTTCTCTGTAATGTTGCATGCCCACCACCTCCTGTTG
AAGGTCAACAGAAAGATCTGAAATGGAATCTTGCCGTTATTCAGCTTTTTTCTGCT
GAAGGAATGGACACGTTTATTCGAGTTCTGCAAAAATTGAACAGTATTCTGACTCA
GCCTTGGAGGCTCCATGTCAACATGGGGACTACCCTTCACAGAGTTACTACTATTT
CAATGGCTCGCTGCACACTCACTCTTCTTAAAACTATGTTAACGGAACTCCTGAGA
GGTGGATCCTTTGAGTTTAAGGACATGCGTGTTCCTTCAGCGCTTGTTACTTTACAT
ATGCTCCTGTGCTCTATCCCCCTCTCAGGTCGTTTGGATAGTGATGAACAGAAAAT
TCAGAATGATATCATTGATATTTTACTGACTTTTACACAAGGAGTTAATGAAAAACT
CACAATCTCAGAAGAGACTCTGGCCAATAATACTTGGTCTTTAATGTTAAAAGAAG
TTCTTTCTTCAATCTTGAAGGTTCCTGAAGGATTTTTTTCTGGACTCATACTCCTTT
CAGAGCTGCTGCCTCTTCCATTGCCCATGCAAACAACTCAGGTTATTGAGCCACAT
GATATATCAGTGGCACTCAACACCCGAAAATTGTGGAGCATGCACCTTCATGTTCA
AGCAAAGTTGCTCCAAGAAATAGTTCGCTCTTTCTCTGGCACAACCTGCCAGCCC
ATTCAACATATGTTACGGCGTATTTGTGTTCAATTGTGTGACCTTGCCTCACCAACT
GCACTTCTGATTATGAGAACTGTGTTGGATTTGATTGTAGAAGACTTGCAAAGCAC
TTCAGAAGATAAAGAAAAACAGTATACTAGCCAAACCACCAGGTTGCTTGCTCTT
CTTGATGCTCTGGCTTCACACAAAGCTTGTAAATTAGCTATTTTGCATCTAATTAAT
GGAACTATTAAAGGTGATGAAAGATATGCAGAGATATTCCAGGATCTTTTAGCTTT

GGTGCGGTCTCCTGGAGACAGTGTTATTCGCCAACAGTGTGTTGAATATGTCACAT
CCATTTTGCAGTCTCTCTGTGATCAGGACATTGCACTTATCTTACCAAGCTCTTCTG
AAGGTTCTATTTCTGAACTGGAGCAGCTCTCCAATTCTCTACCAAATAAAGAATTG
ATGACCTCAATCTGTGACTGTCTGTTGGCTACGCTAGCTAACTCTGAGAGCAGTTA
CAACTGTTTACTGACATGTGTCAGAACAATGATGTTTCTTGCAGAGCATGATTATG
GATTATTTCATTTAAAAAGTTCTTTAAGGAAAAACAGTAGTGCTCTGCATAGTTTAC
TGAAACGAGTGGTCAGCACATTTAGTAAGGACACAGGAGAGCTTGCATCTTCATT
TTTAGAATTTATGAGACAAATTCTTAACTCTGACACAATTGGATGCTGTGGAGATG
ATAATGGTCTCATGGAAGTAGAGGGAGCTCATACATCACGGACGATGAGTATTAAT
GCTGCAGAGTTAAAACAGCTTCTACAAAGCAAAGAAGAAAGTCCAGAAAATTTG
TTCCTTGAACTAGAGAAGCTTGTTTTGGAACATTCAAAAGATGATGACAATCTGGA
TTCTTTGTTGGACAGTGTAGTTGGACTTAAGCAGATGCTGGAGTCATCAGGTGACC
CTTTACCTCTCAGTGACCAGGATGTAGAACCAGTACTTTCAGCTCCAGAATCTCTT
CAGAATCTGTTTAACAATAGGACTGCCTATGTGCTTGCTGATGTCATGGATGATCAG
TTGAAATCTATGTGGTTCACTCCATTTCAGGCTGAAGAGATAGATACAGATCTGGA
TTTGGTAAAGGTTGACTTAATTGAACTCTCTGAAAAATGCTGTAGTGACTTTGATT
TGCACTCAGAATTAGAGCGCTCATTTTTGTCAGAACCATCATCTCCAGGAAGAACC
AAGACTACTAAAGGATTCAAACTTGGGAAGCACAAGCATGAGACCTTTATAACGT
CAAGTGGAAAATCTGAATACATTGAACCTGCCAAAAGAGCTCATGTTGTGCCACC
ACCAAGAGGAAGGGGCAGGGGAGGATTTGGACAGGGTATACGACCTCATGATATT
TTTCGTCAGAGAAAACAGAACACAAGTAGACCACCATCTATGCATGTGGATGACT
TTGTTGCTGCTGAAAGTAAAGAAGTGGTTCCTCAAGATGGAATACCTCCACCAAA
ACGGCCACTCAAAGTATCACAGAAGATTTCTTCCCGTGGTGGGTTTTCAGGCAATA
GAGGAGGACGGGGTGCTTTCCACAGTCAGAATAGGTTTTTCACACCACCTGCTTC
AAAAGGAAACTACAGTCGTCGGGAAGGAACAAGAGGCTCCAGTTGGAGTGCTCA
GAATACTCCTCGAGGAAATTACAATGAAAGTCGTGGAGGCCAGAGCAATTTTAAC
AGAGGCCCTCTTCCACCATTACGACCCCTTAGTTCTACAGGTTACCGCCCAAGTCC
TCGGGACCGTGCTTCTAGAGGTCGTGGGGGACTTGGACCTTCCTGGGCTAGTGCA
AATAGCGGCAGTGGAGGCTCAAGAGGAAAGTTTGTTAGTGGAGGCAGTGGTAGA
GGTCGTCATGTACGCTCCTTTACACGATAA

Amino acid sequence (1812 aa) (C-term 1131–1812 aa in red)
MAVDSAMELLFLDTFKHPSAEQSSHIDVVRFPCVVYINEVRVIPPGVRAHSSLPDNRA
YGETSPHTFQLDLFFNNVSKPSAPVFDRLGSLEYDENTSIIFRPNSKVNTDGLVLRGW
YNCLTLAIYGSVDRVISHDRDSPPPPPPPPPPQPQPSLKRNPKHADGEKEDQFNGSPP
RPQPRGPRTPPGPPPPDDDEDDPVPLPVSGDKEEDAPHREDYFEPISPDRNSVPQEGQ
YSDEGEVEEEQQEEGEEDEDDVDVEEEEDEDEDDRRTVDSIPEEEEEDEEEEGEEDEE
GEGDDGYEQISSDEDGIADLERETFKYPNFDVEYTAEDLASVPPMTYDPYDRELVPLL
YFSCPYKTTFEIEISRMKDQGPDKENSGAIEASVKLTELLDLYREDRGAKWVTALEEIP
SLIIKGLSYLQLKNTKQDSLGQLVDWTMQALNLQVALRQPIALNVRQLKAGTKLVSS
LAECGAQGVTGLLQAGVISGLFELLFADHVSSSLKLNAFKALDSVISMTEGMEAFLR
GRQNEKSGYQKLLELILLDQTVRVVTAGSAILQKCHFYEVLSEIKRLGDHLAEKTSSL
PNHSEPDHDTDAGLERTNPEYENEVEASMDMDLLESSNISEGEIERLINLLEEVFHLM
ETAPHTMIQQPVKSFPTMARITGPPERDDPYPVLFRYLHSHHFLELVTLLLSIPVTSAH

PGVLQATKDVLKFLAQSQKGLLFFMSEYEATNLLIRALCHFYDQDEEEGLQSDGVID
DAFALWLQDSTQTLQCITELFSHFQRCTASEETDHSDLLGTLHNLYLITFNPVGRSAVG
HVFSLEKNLQSLITLMEYYSKEALGDSKSKKSVAYNYACILILVVVQSSSDVQMLEQH
AASLLKLCKADENNAKLQELGKWLEPLKNLRFEINCIPNLIEYVKQNIDNLMTPEGV
GLTTALRVLCNVACPPPPVEGQQKDLKWNLAVIQLFSAEGMDTFIRVLQKLNSILTQP
WRLHVNMGTTLHRVTTISMARCTLTLLKTMLTELLRGGSFEFKDMRVPSALVTLHM
LLCSIPLSGRLDSDEQKIQNDIIDILLTFTQGVNEKLTISEETLANNTWSLMLKEVLSSIL
KVPEGFFSGLILLSELLPLPLPMQTTQVIEPHDISVALNTRKLWSMHLHVQAKLLQEIV
RSFSGTTCQPIQHMLRRICVQLCDLASPTALLIMRTVLDLIVEDLQSTSEDKEKQYTSQ
TTRLLALLDALASHKACKLAILHLINGTIKGDERYAEIFQDLLALVRSPGDSVIRQQCV
EYVTSILQSLCDQDIALILPSSSEGSISELEQLSNSLPNKELMTSICDCLLATLANSESSY
NCLLTCVRTMMFLAEHDYGLFHLKSSLRKNSSALHSLLKRVVSTFSKDTGELASSFLE
FMRQILNSDTIGCCGDDNGLMEVEGAHTSRTMSINAAELKQLLQSKEESPENLFLELE
KLVLEHSKDDDNLDSLLDSVVGLKQMLESSGDPLPLSDQDVEPVLSAPESLQNLFNN
RTAYVLADVMDDQLKSMWFTPFQAEEIDTDLDLVKVDLIELSEKCCSDFDLHSELER
SFLSEPSSPGRTKTTKGFKLGKHKHETFITSSGKSEYIEPAKRAHVVPPPRGRGRGGFG
QGIRPHDIFRQRKQNTSRPPSMHVDDFVAAESKEVVPQDGIPPPKRPLKVSQKISSRG
GFSGNRGGRGAFHSQNRFFTPPASKGNYSRREGTRGSSWSAQNTPRGNYNESRGGQ
SNFNRGPLPPLRPLSSTGYRPSPRDRASRGRGGLGPSWASANSGSGGSRGKFVSGGSG
RGRHVRSFTR

Human *VIRMA* N-terminal (KIAA1429 Isoform 2)

Nucleotide sequence: 3444 nt
ATGGCGGTGGACTCGGCGATGGAGCTGTTATTTTTAGATACTTTTAAACACCCGAG
CGCTGAGCAAAGTTCTCATATAGATGTGGTTCGTTTTCCATGTGTGGTTTATATCAA
TGAAGTCCGAGTCATACCCCCAGGAGTAAGAGCCCATAGCAGTCTGCCAGACAAT
AGAGCATATGGAGAGACATCTCCCCATACATTTCAATTAGACTTATTCTTCAACAAT
GTAAGCAAACCAAGTGCCCCTGTTTTCGATAGGTTGGGAAGCCTGGAATATGATG
AGAATACTTCCATCATCTTTAGACCTAACTCAAAGGTGAATACTGATGGTCTGGTG
CTAAGAGGCTGGTATAACTGTCTGACACTGGCAATATATGGATCAGTGGATAGAGT
GATAAGTCATGACAGAGACTCTCCACCACCACCACCTCCACCGCCACCACCTCCC
CAGCCACAACCAAGTTTGAAAAGGAATCCAAAACATGCTGATGGGGAGAAAGAA
GATCAGTTTAATGGAAGCCCTCCAAGACCACAGCCAAGGGGACCAAGAACTCCTC
CAGGACCCCCTCCACCTGATGATGATGAAGATGATCCTGTGCCTCTGCCAGTGTCT
GGTGACAAGGAAGAGGATGCTCCTCATAGAGAAGATTACTTTGAGCCCATTTCTC
CTGATCGGAATTCTGTTCCCCAGGAAGGGCAATATTCTGATGAAGGAGAAGTAGA
AGAGGAACAACAAGAAGAAGGAGAAGAAGATGAAGATGATGTGGATGTAGAGG
AAGAAGAGGATGAGGATGAGGATGATCGACGAACAGTAGACAGTATTCCTGAGG
AGGAAGAGGAAGATGAAGAGGAAGAAGGTGAAGAGGATGAAGAAGGTGAAGGG
GATGATGGTTATGAACAAATTTCCAGTGATGAAGATGGAATTGCTGACTTGGAACG
TGAAACATTTAAGTATCCAAACTTTGATGTTGAATACACTGCTGAAGACTTAGCTT
CAGTTCCTCCTATGACATATGATCCATATGACAGGGAGCTTGTACCACTCTTATACT
TCAGTTGTCCATACAAGACTACTTTTGAAATTGAAATCAGTAGAATGAAGGATCAA

GGTCCAGATAAAGAAAATTCAGGGGCAATCGAAGCCTCAGTGAAGTTAACAGAA
CTCTTAGATTTGTATAGAGAAGATAGAGGTGCAAAATGGGTAACAGCTTTAGAAGA
AATTCCAAGTTTAATAATAAAAGGGTTAAGCTATTTGCAATTGAAAAACACAAAAC
AAGACTCCCTTGGCCAGTTGGTAGACTGGACCATGCAAGCTTTAAATTTACAAGTA
GCGCTTCGCCAACCTATCGCCTTAAATGTTCGACAGCTCAAAGCTGGACCAAATT
AGTGTCCTCACTAGCAGAATGTGGGGCTCAAGGAGTTACAGGACTGCTACAAGCA
GGAGTGATCAGTGGATTATTTGAACTTCTGTTTGCTGATCACGTATCATCTTCTCTT
AAGTTAAATGCTTTTAAAGCTTTGGACAGTGTCATTAGTATGACAGAAGGAATGGA
AGCTTTTTTAAGAGGTAGGCAGAATGAAAAAAGTGGTTATCAAAAGCTTCTGGAA
CTCATACTTTTAGATCAGACTGTGAGGGTTGTTACTGCTGGTTCAGCTATTCTCCAA
AAATGCCATTTCTATGAAGTCTTGTCAGAGATTAAAAGACTTGGTGACCATTTAGC
AGAGAAGACTTCATCTCTTCCTAACCACAGTGAACCTGATCACGACACAGATGCT
GGACTTGAGAGAACAAACCCAGAATATGAAAATGAGGTGGAAGCTTCTATGGATA
TGGATCTTTTGGAATCCTCAAATATAAGTGAAGGGGAAATAGAAAGGCTTATTAAC
CTCCTAGAAGAAGTTTTTCATTTAATGGAAACTGCCCCTCATACAATGATCCAACA
ACCTGTTAAGTCTTTCCCAACGATGGCACGAATTACTGGACCTCCAGAGAGGGAT
GATCCATACCCTGTTCTCTTTAGATATCTTCACAGTCATCACTTCTTGGAGTTGGTT
ACCTTGCTTCTGTCAATTCCAGTAACAAGTGCTCACCCTGGTGTGCTGCAAGCCAC
AAAAGATGTTTTGAAGTTTCTTGCACAGTCACAGAAGGGTCTTCTTTTTTTTATGT
CGGAATATGAAGCAACAAATTTATTGATCCGAGCTCTGTGTCACTTTTATGATCAAG
ATGAGGAGGAAGGTCTCCAATCTGATGGTGTTATTGATGATGCATTTGCCTTGTGG
CTACAGGACTCAACACAGACATTGCAATGTATTACAGAACTGTTCAGCCATTTTCA
GCGTTGTACAGCCAGTGAAGAAACAGACCATTCAGATCTCTTGGGAACCCTGCAC
AATCTTTATTTGATTACTTTTAATCCTGTGGGAAGATCAGCTGTTGGCCATGTTTTTA
GTCTGGAGAAAAATCTCCAAAGTCTTATTACTCTAATGGAGTACTATTCCAAAGAA
GCCTTGGGTGATTCCAAATCTAAGAAGTCAGTAGCTTATAATTACGCATGCATACTT
ATTTTGGTGGTGGTTCAGTCTTCCAGTGATGTTCAAATGCTAGAACAACATGCAGC
ATCTCTCTTGAAGCTTTGTAAAGCAGATGAAAATAATGCTAAATTGCAAGAACTTG
GCAAGTGGCTTGAACCTCTGAAAAACCTTAGATTTGAAATTAACTGCATCCCAAA
CTTAATTGAGTATGTTAAGCAGAATATCGATAACTTGATGACCCCAGAAGGAGTTG
GCCTTACCACTGCCTTACGTGTTCTCTGTAATGTTGCATGCCCACCACCTCCTGTTG
AAGGTCAACAGAAAGATCTGAAATGGAATCTTGCCGTTATTCAGCTTTTTTCTGCT
GAAGGAATGGACACGTTTATTCGAGTTCTGCAAAAATTGAACAGTATTCTGACTCA
GCCTTGGAGGCTCCATGTCAACATGGGGACTACCCTTCACAGAGTTACTACTATTT
CAATGGCTCGCTGCACACTCACTCTTCTTAAAACTATGTTAACGGAACTCCTGAGA
GGTGGATCCTTTGAGTTTAAGGACATGCGTGTTCCTTCAGCGCTTGTTACTTTACAT
ATGCTCCTGTGCTCTATCCCCCTCTCAGGTCGTTTGGATAGTGATGAACAGAAAAT
TCAGAATGATATCATTGATATTTTACTGACTTTTACACAAGGAGTTAATGAAAAACT
CACAATCTCAGAAGAGACTCTGGCCAATAATACTTGGTCTTTAATGTTAAAAGAAG
TTCTTTCTTCAATCTTGAAGGTTCCTGAAGGATTTTTTTCTGGACTCATACTCCTTT
CAGAGCTGCTGCCTCTTCCATTGCCCATGCAAACAACTCAGGTATCACTTCCATAT
AACATGCATCTTATAAATGACTGCAGTAACACTTTTTAA

Amino acid sequence(1147 aa)

MAVDSAMELLFLDTFKHPSAEQSSHIDVVRFPCVVYINEVRVIPPGVRAHSSLPDNRA
YGETSPHTFQLDLFFNNVSKPSAPVFDRLGSLEYDENTSIIFRPNSKVNTDGLVLRGW
YNCLTLAIYGSVDRVISHDRDSPPPPPPPPPPPQPQPSLKRNPKHADGEKEDQFNGSPP
RPQPRGPRTPPGPPPPDDDEDDPVPLPVSGDKEEDAPHREDYFEPISPDRNSVPQEGQ
YSDEGEVEEEQQEEGEEDEDDVDVEEEEDEDEDDRRTVDSIPEEEEEDEEEEGEEDEE
GEGDDGYEQISSDEDGIADLERETFKYPNFDVEYTAEDLASVPPMTYDPYDRELVPLL
YFSCPYKTTFEIEISRMKDQGPDKENSGAIEASVKLTELLDLYREDRGAKWVTALEEIP
SLIIKGLSYLQLKNTKQDSLGQLVDWTMQALNLQVALRQPIALNVRQLKAGTKLVSS
LAECGAQGVTGLLQAGVISGLFELLFADHVSSSLKLNAFKALDSVISMTEGMEAFLR
GRQNEKSGYQKLLELILLDQTVRVVTAGSAILQKCHFYEVLSEIKRLGDHLAEKTSSL
PNHSEPDHDTDAGLERTNPEYENEVEASMDMDLLESSNISEGEIERLINLLEEVFHLM
ETAPHTMIQQPVKSFPTMARITGPPERDDPYPVLFRYLHSHHFLELVTLLLSIPVTSAH
PGVLQATKDVLKFLAQSQKGLLFFMSEYEATNLLIRALCHFYDQDEEEGLQSDGVID
DAFALWLQDSTQTLQCITELFSHFQRCTASEETDHSDLLGTLHNLYLITFNPVGRSAVG
HVFSLEKNLQSLITLMEYYSKEALGDSKSKKSVAYNYACILILVVVQSSSDVQMLEQH
AASLLKLCKADENNAKLQELGKWLEPLKNLRFEINCIPNLIEYVKQNIDNLMTPEGV
GLTTALRVLCNVACPPPPVEGQQKDLKWNLAVIQLFSAEGMDTFIRVLQKLNSILTQP
WRLHVNMGTTLHRVTTISMARCTLTLLKTMLTELLRGGSFEFKDMRVPSALVTLHM
LLCSIPLSGRLDSDEQKIQNDIIDILLTFTQGVNEKLTISEETLANNTWSLMLKEVLSSIL
KVPEGFFSGLILLSELLPLPLPMQTTQVSLPYNMHLINDCSNTF

Human *ZC3H13* full length (ZC3H13 Isoform 1, NM_001076788.1)

Nucleotide sequence: 5010 nt (Sequences in blue and in red are defined as *ZC3H13* N-term and C-term)

ATGTCAAAAATTAGAAGGAAGGTCACAGTGGAAAATACCAAGACTATATCTGAT
AGCACATCCCGAAGACCCAGTGTATTTGAGAGGCTTGGACCCAGCACTGGCAGT
ACAGCAGAGACACAGTGCCGTAACTGGCTGAAGACTGGCAACTGCCTCTATGGA
AACACATGTAGATTCGTACATGGCCCTTCACCTCGTGGTAAAGGTTATAGCAGCA
ATTATAGAAGGTCACCAGAAAGACCTACAGGGGATCTTAGAGAAAGAATGAAG
AACAAGCGCCAAGACGTGGACACTGAGCCCCAGAAACGAAATACAGAGGAGTC
ATCCTCACCTGTTAGGAAAGAATCTTCAAGAGGGAGACATAGGGAAAAGGAAGA
CATAAAAATCACTAAGGAAAGAACTCCAGAAAGTGAAGAAGAAAATGTAGAAT
GGGAAACTAATAGAGATGATTCTGACAATGGAGATATTAATTATGATTATGTTCA
TGAATTGTCATTGGAAATGAAGCGTCAGAAGATACAGAGGGAATTAATGAAGCT
GGAACAAGAAAACATGGAGAAGAGAGAAGAAATTATCATTAAAAAGGAGGTTT
CACCAGAAGTGGTTAGATCAAAATTGTCCCCGTCACCTTCTCTAAGAAAGTCTAG
CAAATCTCCGAAGCGAAAATCAAGCCCGAAGTCGTCTTCAGCTAGCAAGAAAGA
TAGGAAGACATCTGCAGTATCTTCTCCCCTGTTGGACCAGCAGAGAAATTCAAAA
ACCAACCAAAGTAAAAAGAAAGGACCACGTACTCCTAGTCCACCCCCTCCTATA
CCAGAAGATATCGCTCTGGGGAAAAAATACAAAGAAAAATATAAAGTAAAAGA
CAGGATAGAAGAAAAAACAAGAGATGGAAAGGACAGAGGACGAGATTTTGAAC

GACAAAGAGAAAAGAGAGACAAGCCAAGGTCTACTTCCCCAGCAGGACAGCAT
CATTCTCCTATATCTTCTAGACATCACTCATCTTCCTCACAATCAGGATCATCTAT
TCAAAGACATTCTCCTTCTCCTCGTCGAAAAAGAACTCCTTCACCATCTTATCAG
CGGACACTAACTCCACCTTTACGACGCTCTGCCTCTCCTTATCCTTCACATTCTTT
GTCGTCTCCCCAGAGAAAGCAGAGTCCTCCAAGACATCGCTCTCCAATGCGAGA
GAAAGGGAGACATGATCATGAACGAACTTCACAGTCTCATGATCGACGCCATGA
AAGGAGGGAAGATACTAGGGGCAAACGAGACAGAGAAAAGGACTCAAGAGAA
GAACGAGAATATGAACAGGATCAGAGCTCTTCTAGAGACCACAGAGATGACAGA
GAACCTCGAGATGGTCGGGATCGGAGAGATGCCAGAGATACTAGGGACCGAAG
GGAACTAAGAGACTCCAGAGACATGCGGGACTCAAGGGAGATGAGAGATTATA
GCAGAGATACCAAAGAGAGCCGTGATCCCAGAGATTCTCGGTCCACTCGTGATG
CCCATGACTACAGGGACCGTGAAGGTCGAGATACTCATCGAAAGGAGGATACAT
ATCCAGAAGAATCCCGGAGTTATGGCCGAAACCATTTGAGAGAAGAAAGTTCTC
GTACGGAAATAAGGAATGAGTCCAGAAATGAGTCTCGAAGTGAAATTAGAAATG
ACCGAATGGGCCGAAGTAGGGGGAGGGTTCCTGAGTTACCTGAAAAGGGAAGTC
GAGGCTCAAGAGGTTCTCAAATTGATAGTCACAGTAGTAATAGCAACTATCATG
ACAGCTGGGAAACTCGAAGTAGCTATCCTGAAAGAGATAGATATCCTGAAAGAG
ACAACAGAGATCAAGCAAGGGATTCTTCCTTTGAGAGAAGACATGGAGAGCGAG
ACCGTCGTGACAACAGAGAGAGAGATCAAAGACCAAGCTCACCAATTCGACATC
AGGGAAGGAATGACGAGCTTGAGCGTGATGAAAGAAGAGAGGAACGAAGAGTA
GACAGAGTGGATGATAGGAGAGATGAAAGGGCTAGAGAGAGAGATCGGGAACG
AGAACGAGACAGGGAGCGGGAGAGAGAGAGGGAACGTGAACGGGATCGGGAA
AGAGAAAAAGAGAGAGAACTAGAAAGAGAGCGTGCTAGGGAACGGGAGAGAG
AAAGAGAAAAAGAGAGAGATCGTGAAAGGGATAGAGACCGAGACCACGATCGA
GAGCGGGAAAGAGAGAGGGAACGAGACAGGGAAAAAGAACGGGAACGAGAAA
GAGAAGAGAGAGAGAGGGAGAGAGAGCGAGAACGGGAGAGAGAGCGAGAGCG
AGAACGGGAACGAGAAAGAGCGAGAGAAAGGGATAAAGAACGAGAACGCCAA
AGGGATTGGGAAGACAAAGACAAAGGACGAGATGACCGCAGAGAAAAGCGAGA
AGAGATCCGAGAAGATAGGAATCCAAGAGATGGACATGATGAAAGAAAATCAA
AGAAGCGCTATAGAAATGAAGGGAGTCCCAGCCCTAGACAGTCCCCGAAGCGCC
GGCGTGAACATTCTCCGGACAGTGATGCCTACAACAGTGGAGATGATAAAAATG
AAAAACACAGACTCTTGAGCCAAGTTGTACGACCTCAAGAATCTCGTTCTCTTAG
TCCCTCGCACCTCACAGAAGACAGACAGGGTAGATGGAAAGAGGAGGATCGTAA
ACCAGAAAGGAAAGAGAGTTCAAGGCGCTACGAAGAACAGGAACTCAAGGAGA
AAGTTTCTTCTGTAGATAAACAGAGAGAACAGACAGAAATCCTGGAAAGCTCAA
GAATGCGTGCACAGGACATTATAGGACACCACCAGTCTGAAGATCGAGAGACAT
CTGATCGAGCTCATGATGAAAACAAGAAGAAAGCAAAAATTCAAAAGAAACCA
ATTAAGAAAAAGAAAGAGGATGATGTTGGAATAGAGAGGGGTAACATAGAGAC
AACATCTGAAGATGGTCAAGTATTTTCACCAAAAAAAGGACAGAAAAAGAAAA
GCATTGAAAAAAAACGTAAAAAATCCAAAGGTGATTCTGATATTTCTGATGAAG
AAGCAGCCCAGCAAAGTAAGAAGAAAAGAGGCCCACGGACTCCCCCTATAACA
ACTAAAGAGGAATTGGTTGAAATGTGCAATGGTAAGAATGGTATTCTAGAGGAC
TCCCAGAAAAAAGAAGATACAGCATTCAGTGACTGGTCTGATGAGGATGTCCCT
GACCGTACAGAGGTGACAGAAGCAGAGCATACTGCCACCGCCACGACTCCTGGT

AGTACCCCTTCTCCTCTATCTTCTCTTCTTCCTCCTCCACCGCCTGTGGCTACTGCC
ACTGCTACAACTGTGCCTGCAACTCTTGCTGCCACTACTGCTGCTGCCGCCACCT
CTTTCAGCACATCTGCCATCACTATTTCCACCTCTGCCACCCCCACCAATACCACC
AATAATACTTTTGCCAATGAAGACTCACACAGAAAATGCCACAGAACACGAGTA
GAAAAAGTAGAGACGCCTCACGTGACTATAGAAGATGCACAGCATCGCAAGCCT
ATGGATCAAAAGAGGAGCAGCAGCCTCGGGAGCAATCGGAGTAACCGTAGTCAT
ACGTCTGGTCGTCTTCGCTCCCCATCCAATGATTCAGCCCATCGAAGTGGAGATG
ACCAAAGTGGTCGAAAGAGAGTACTGCACAGTGGCTCAAGAGATAGAGAAAAA
ACAAAAAGCCTGGAAATCACAGGAGAGAGAAAATCTAGGATTGATCAGTTAAA
GCGTGGAGAACCCAGTCGAAGTACTTCTTCAGATCGCCAGGATTCAAGAAGCCA
TAGTTCAAGAAGAAGTTCTCCAGAGTCAGATCGACAGGTCCATTCAAGATCTGG
GTCATTTGATAGCAGAGACAGGCTTCAAGAACGAGATCGATATGAACACGACAG
AGAGCGCGAGAGAGAGAGGAGAGATACGAGGCAGAGAGAATGGGACCGAGAT
GCTGATAAAGATTGGCCACGCAACAGGGATCGAGATAGATTGCGAGAACGAGA
ACGAGAGAGAGAACGAGACAAAAGGAGAGACTTGGATAGGGAAAGAGAGAGA
CTAATTTCTGATTCTGTTGAAAGGGACAGGGACAGAGACAGAGACAGAACTTTT
GAGAGTTCTCAAATAGAGTCTGTGAAACGCTGTGAAGCAAAACTGGAAGGTGAA
CATGAAAGGGATCTAGAAAGCACTTCCCGAGACTCTCTAGCCTTGGATAAAGAG
AGAATGGATAAAGATCTGGGATCTGTGCAGGGATTTGAAGAAACAAATAAATCC
GAGAGAACTGAGAGTCTGGAAGCAGGAGATGACGAGTCCAAGTTAGATGATGC
ACATTCATTAGGCTCTGGTGCTGGAGAAGGATACGAGCCAATCAGTGATGACGA
ACTAGATGAAATTCTGGCAGGTGATGCAGAAAAGAGGGAGGACCAACAGGATG
AGGAGAAGATGCCAGATCCCTTAGATGTGATAGATGTGGATTGGTCTGGTCTTAT
GCCAAAGCATCCAAAAGAACCACGAGAGCCTGGGGCTGCACTCTTAAAATTCAC
ACCTGGAGCTGTTATGCTAAGAGTTGGGATTTCTAAAAAGTTGGCAGGTTCTGAA
CTCTTTGCCAAAGTCAAAGAAACATGTCAGAGACTTTTAGAAAAACCCAAAGAT
GCAGACAATCTCTTTGAACATGAATTGGGGGCTCTCAATATGGCTGCATTACTAC
GAAAAGAAGAAAGAGCAAGTCTTCTTAGTAATCTTGGCCCATGTTGTAAGGCGTT
GTGCTTCAGACGGGATTCTGCAATTCGAAAGCAGCTTGTTAAAAATGAGAAGGG
CACCATAAAACAAGCTTACACGAGTGCTCCAATGGTAGACAATGAATTACTTCG
ATTGAGTCTTCGGTTATTTAAGCGGAAGACTACTTGCCATGCTCCAGGACATGAA
AAGACTGAAGATAATAAACTTTCACAGTCCAGTATCCAACAGGAACTGTGTGTG
TCTTAA

Amino acid sequence (1669 aa) (N-term 1-1106 aa in blue, C-term 1107–1669 aa in red)

MSKIRRKVTVENTKTISDSTSRRPSVFERLGPSTGSTAETQCRNWLKTGNCLYGNTC
RFVHGPSPRGKGYSSNYRRSPERPTGDLRERMKNKRQDVDTEPQKRNTEESSSPVRK
ESSRGRHREKEDIKITKERTPESEEENVEWETNRDDSDNGDINYDYVHELSLEMKRQ
KIQRELMKLEQENMEKREEIIIKKEVSPEVVRSKLSPSPSLRKSSKSPKRKSSPKSSSAS
KKDRKTSAVSSPLLDQQRNSKTNQSKKKGPRTPSPPPPIPEDIALGKKYKEKYKVKD
RIEEKTRDGKDRGRDFERQREKRDKPRSTSPAGQHHSPISSRHHSSSSQSGSSIQRHSP
SPRRKRTPSPSYQRTLTPPLRRSASPYPSHSLSSPQRKQSPPRHRSPMREKGRHDHERT
SQSHDRRHERREDTRGKRDREKDSREEREYEQDQSSSRDHRDDREPRDGRDRRDAR
DTRDRRELRDSRDMRDSREMRDYSRDTKESRDPRDSRSTRDAHDYRDREGRDTHR

KEDTYPEESRSYGRNHLREESSRTEIRNESRNESRSEIRNDRMGRSRGRVPELPEKGS
RGSRGSQIDSHSSNSNYHDSWETRSSYPERDRYPERDNRDQARDSSFERRHGERDRR
DNRERDQRPSSPIRHQGRNDELERDERREERRVDRVDDRRDERARERDRERERDRE
RERERERERDREREKERELERERARERERERERERDKERDRERDRDRDHDRERERERERDR
EKEREREREERERERERERERERERERERERERERARERDKERERQRDWEDKDKGRDDRR
EKREEIREDRNPRDGHDERKSKKRYRNEGSPSPRQSPKRRREHSPDSDAYNSGDDKN
EKHRLLSQVVRPQESRSLSPSHLTEDRQGRWKEEDRKPERKESSRRYEEQELKEKVS
SVDKQREQTEILESSRMRAQDIIGHHQSEDRETSDRAHDENKKKAKIQKKPIKKKKE
DDVGIERGNIETTSEDGQVFSPKKGQKKKSIEKKRKKSKGDSDISDEEAAQQSKKKR
GPRTPPITTKEELVEMCNGKNGILEDSQKKEDTAFSDWSDEDVPDRTEVTEAEHTAT
ATTPGSTPSPLSSLLPPPPPVATATATTVPATLAATTAAAATSFSTSAITISTSATPTNTT
NNTFANEDSHRKCHRTRVEKVETPHVTIEDAQHRKPMDQKRSSSLGSNRSNRSHTSG
RLRSPSNDSAHRSGDDQSGRKRVLHSGSRDREKTKSLEITGERKSRIDQLKRGEPSRS
TSSDRQDSRSHSSRRSSPESDRQVHSRSGSFDSRDRLQERDRYEHDRERERERRDTRQ
REWDRDADKDWPRNRDRDRLRERERERERDKRRDLDRERERLISDSVERDRDRDR
DRTFESSQIESVKRCEAKLEGEHERDLESTSRDSLALDKERMDKDLGSVQGFEETNK
SERTESLEAGDDESKLDDAHSLGSGAGEGYEPISDDELDEILAGDAEKREDQQDEEK
MPDPLDVIDVDWSGLMPKHPKEPREPGAALLKFTPGAVMLRVGISKKLAGSELFAK
VKETCQRLLEKPKDADNLFEHELGALNMAALLRKEERASLLSNLGPCCKALCFRRD
SAIRKQLVKNEKGTIKQAYTSAPMVDNELLRLSLRLFKRKTTCHAPGHEKTEDNKLS
QSSIQQELCVS

Supplementary table legends

Table S1 Proteomic data of METTL3, METTL14, WTAP, and VIRMA

Table S2 Brief summary of high throughput sequencing data

Table S3 Examples for m^{6}A peak annotations in *VIRMA*$^{mut/-}$ and control samples

Table S4 The list of whole transcriptome showing the changes of m^{6}A peak and expression level between *VIRMA*$^{mut/-}$ and control samples

Table S5 Summary of upregulated m^{6}A-containing genes with change of expression more than 2 folds by comparing *VIRMA*$^{mut/-}$ with control

Table S6 Summary of GO enrichment network of differentially expressed m^{6}A-containing genes

Table S7 Summary of VIRMA RIP targets and its overlap with m^{6}A-seq targets

Table S8 Lists of genes showing mRNA 3`UTR length change under depletion of VIRMA, METTL3 and CPSF5 versus control

Table S9 Primers and siRNA sequences used in this work

Table S10 Antibodies used in this work
